# Gut microbiota composition and gene expression changes induced in the *Apis cerana* exposed to acetamiprid and difenoconazole at environmentally realistic concentrations alone or combined

**DOI:** 10.3389/fphys.2023.1174236

**Published:** 2023-05-03

**Authors:** Wensu Han, Zheyuan Ye, Yifan Gu, Yihai Zhong, Jinglin Gao, Shan Zhao, Shijie Wang

**Affiliations:** ^1^ Environment and Plant Protection Institute, Chinese Academy of Tropical Agricultural Sciences, Haikou, China; ^2^ Bee Industry Technology Research Center, Chinese Academy of Tropical Agricultural Sciences, Haikou, China; ^3^ Sanya Institute of China Agricultural University, Sanya, China; ^4^ Department of Entomology, College of Plant Protection, China Agricultural University, Beijing, China

**Keywords:** *Apis cerana*, acetamiprid, difenoconazole, gut microbiota, gene expression

## Abstract

*Apis cerana* is an important pollinator of agricultural crops in China. In the agricultural environment, *A. cerana* may be exposed to acetamiprid (neonicotinoid insecticide) and difenoconazole (triazole fungicide), alone or in combination because they are commonly applied to various crops. At present, our understanding of the toxicological effects of acetamiprid and difenoconazole on honey bee gut microbiomes is limited. The primary objective of this study was to explore whether these two pesticides affect honey bees’ gut microbiota and to analyze the transcriptional effects of these two pesticides on honey bees’ head and gut. In this study, adults of *A. cerana* were exposed to acetamiprid and/or difenoconazole by contaminated syrup at field-realistic concentrations for 10 days. Results indicated that acetamiprid and/or difenoconazole chronic exposure did not affect honey bees’ survival and food consumption, whereas difenoconazole decreased the weight of honey bees. 16S rRNA sequencing suggested that difenoconazole and the mixture of difenoconazole and acetamiprid decreased the diversity index and shaped the composition of gut bacteria microbiota, whereas acetamiprid did not impact the gut bacterial community. The ITS sequence data showed that neither of the two pesticides affected the fungal community structure. Meanwhile, we also observed that acetamiprid or difenoconazole significantly altered the expression of genes related to detoxification and immunity in honey bees’ tissues. Furthermore, we observed that the adverse effect of the acetamiprid and difenoconazole mixture on honey bees’ health was greater than that of a single mixture. Taken together, our study demonstrates that acetamiprid and/or difenoconazole exposure at field-realistic concentrations induced changes to the honey bee gut microbiome and gene expression.

## Introduction

*Apis cerana* is an important indigenous species, and it was the keystone pollinator of agricultural crops in China before the introduction of European honeybees *Apis mellifera* in the late 19th century (Guo et al., 2015). Compared to *A. mellifera*, *A. cerana* has a strong ability to collect scattered floral resources and work in cold temperatures and defend against the ectoparasitic mite, *Varroa destructor* (Chen et al., 2017). The managed colony of *A. cerana* is the main pollinator released in the greenhouse, especially in parts of Southeast Asia (Osterman et al., 2021). Currently, despite the ecological and economic importance, *A. cerana* colonies have undergone substantial range contractions (Gemeda et al., 2017) and a population decline in recent years (Zhao et al., 2017). Previous reports from all over the world showed that exposure to pesticides, in particular, the class of insecticides known as neonicotinoids, is one of the reasons for the population decline of honey bees and wild bees (Pisa et al., 2015; Brandt et al., 2016; Mitchell et al., 2017; Mokkapati et al., 2021; Willis Chan and Raine, 2021).

Neonicotinoids are neurotoxins that act as nicotinic acetylcholine receptor agonists in the insect central nervous system and cause over-stimulations to nerve cells resulting in paralysis and death (Wang et al., 2020). Owing to their lower toxicity to mammals, neonicotinoids began to be widely used in agriculture starting during the 1990s (Matsuda et al., 2020). Global sales of neonicotinoids are worth US$1 billion/year (Tasman et al., 2020). Neonicotinoids are systemically incorporated into plant tissues, including pollen and nectar (David et al., 2016). Pollens and nectars are the major food source for honey bees. Thus, foragers are often directly exposed to neonicotinoid insecticides while visiting flowers, and hive bees can be exposed to contaminated pollens and nectars that are brought back to the hive. Mitchell et al. (2017) reported that 75% of honey bee colony samples from all over the world contain at least one neonicotinoid.

In addition to insecticides, pollen and nectar analyses showed a high incidence of fungicides (Mullin et al., 2010; David et al., 2016; Tong et al., 2016; Gaweł et al., 2019). In comparison, fungicide application exceeds that of both insecticides and herbicides on a global scale (Rondeau and Raine, 2022). Fungicides are commonly applied during the blooming period to control fungal disease, which are assumed to be without risks to pollinators (Mitchell et al., 2017; Favaro et al., 2019). However, much more recent research works have shown that fungicides can affect honey bees’ food consumption (Liao et al., 2017), nest recognition (Artz and Pitts-Singer, 2015), metabolism (Mao et al., 2017; Christen et al., 2019), respiration (Han et al., 2018), and immune function (Degrandi-Hoffman et al., 2015). Moreover, fungicides may enhance the toxicity of insecticides to honey bees, in particular, triazole fungicides and neonicotinoids or pyrethroids in the mixture (Wade et al., 2019).

In agricultural environments, foragers are often exposed to neonicotinoid insecticides and triazole fungicides because these pesticides are frequently co-applied in a tank mix or commonly used in seed coating. All honey bee colony members are chronically exposed to these pesticides through the foraging of contaminated pollens and nectars that are brought back to the colony (Rouzé et al., 2019). Pesticide mixtures can have additive effects through the same or different modes of action or even synergism or antagonism in toxicity. Thus, more attention should be paid to the magnitude of specific mixture-induced effects.

Ingested pesticides are in contact with the honey bee’s gut and may alter its physiology; so, in this study, we investigated the effects of acetamiprid and difenoconazole exposure on the gut microbiota composition and physiology of *A. cerana*, alone or in combination. We selected these compounds due to their systemic properties, which are intensively used throughout the crop growing season and simultaneously detected in pollen and honey samples (Tong et al., 2018; Gaweł et al., 2019).

Acetamiprid belongs to the group of cyano-substituted neonicotinoids, which are generally considered safe for bees, with oral and contact toxicities with two to three orders of magnitude lower than those of the nitro-substituted neonicotinoids, such as imidacloprid, clothianidin, and thiamethoxam (Feyereisen, 2018). Thus, due to the comparatively more “bee-friendly” properties of acetamiprid, it is permitted to be sprayed on flowering crops during daylight when honey bees are actively foraging (Godfray et al., 2014). Mitchell et al. (2017) reported that maximum and average concentrations among positive honey samples were the highest for acetamiprid and thiacloprid, but acetamiprid is present in Asia, whereas thiacloprid is present in Europe.

Difenoconazole belongs to triazole fungicides. This fungicide inhibits sterol biosynthesis, which is crucial for maintaining the cell membrane integrity of fungi (Figueirêdo et al., 2019). Aquatic organisms’ long-term exposure to difenoconazole at low concentrations would result in the bioaccumulation of this compound and elicit estrogenic endocrine-disruption effects (Zhang et al., 2017; Teng et al., 2018). Stingless bee *Tetragonisca angustula* exposed to difenoconazole were less tolerant when it was applied via ingestion or on treated surfaces (Leite et al., 2021). Prado et al. (2020) confirmed that difenoconazole accumulates in tissues of an adult forager of stingless bee *Melipona scutellaris* Latreille, regardless of topical and oral exposure, and caused death. However, according to the PPDB (Pesticide Properties Database, 2019), this fungicide is not considered toxic for bees. Our previous research has shown that the acetamiprid and difenoconazole mixture has greater toxic effects on *A. cerana* than the individual compounds (Han et al., 2017). Hence, intensively studying the impact of these two pesticides on *A. cerana* is imperative.

The gut microbiota is a complex ecosystem of symbiotic bacteria that interacts with multiple organs and systems in the host via metabolites, proteins, and genes. Recently, the gut microbiota has emerged as a critical factor affecting bees’ health and fitness (Ribière et al., 2019). The honey bee’s gut microbiota is relatively simple and conservative; it provides several health benefits to bees such as promoting the digestion of food, stimulating the immune system, protecting against pathogens, increasing the weight in adult bees, and stimulating the expression of host detoxification genes (Zheng et al., 2018; Ribière et al., 2019; Wu et al., 2020). In *A. mellifera*, 95%–99% of gut microbiota is dominated by five to eight core bacterial species. These members belong to different taxa, including *Snodgrassella*, *Gilliamella*, *Bifidobacterium*, *Lactobacillus* Firm-4, *Lactobacillus* Firm-5, *Bartonella*, *Frischella*, and *Commensalibacter* (Motta et al., 2018; Ribière et al., 2019; Motta et al., 2020). In *A. cerana*, the microbial taxa are the same as *A. mellifera* and at a relatively low level, which mainly includes *Bifidobacterium*, *Snodgrassella*, *Gilliamella,* and *Lactobacillus* (Guo et al., 2015). Although several studies have shown that bees’ gut microbiota composition can be disrupted by pesticides (Kakumanu et al., 2016; Motta et al., 2018; Rouzé et al., 2019; Liu et al., 2020; Motta et al., 2020; Zhu et al., 2020) and antibiotic (Raymann et al., 2017) exposure, which have been linked to bees colony mortalities, little is known about the interactive effects of pesticides’ cocktail on the gut, which is one of the main entrances for toxic molecules.

In addition, changes in gene expression were reported in larvae and worker honey bees fed with sublethal doses of neonicotinoid insecticides or fungicides, where genes are related to neurotoxicity, memory formation, stress reaction, metabolism, detoxification, immunity, and other pathways (Christen et al., 2016; Christen and Fent, 2017; Wu et al., 2017; Christen et al., 2019). To the best of our knowledge, little is known about the molecular effects of a mixture of acetamiprid and ergosterol biosynthesis inhibitor (EBI) fungicides (difenoconazole) on honey bee.

A key question in ecotoxicological studies is whether the test doses applied in the laboratory are field realistic (Sgolastra et al., 2018). Therefore, in the present study, we chronically exposed nurse honey bees of *A. cerana* to acetamiprid and difenoconazole via sucrose solution, alone and in combinations. In an attempt to mimic field-realistic conditions, we used the concentrations of acetamiprid and difenoconazole found in beebread (Kubik et al., 2000; Tong et al., 2018; Almasri et al., 2021). Our aim was to evaluate whether the nurse honey bee exposure to acetamiprid and difenoconazole separately and in binary mixtures impacts the abundance and composition of the gut microbiota and whether the transcriptional responses of genes are related to detoxification enzymes, antioxidative enzymes, immune system, neuronal signaling, and development. We focus on target genes that play an important role in the physiology of bees that were previously suggested to be involved in the stress response to pesticides (Christen and Fent, 2017; Christen et al., 2019).

## Materials and methods

### Chemicals and solutions

Standards with a high purity level (98.2% and 96% for acetamiprid and difenoconazole, respectively) were obtained from Hainan Boswell Agrichemical Co., Ltd. The stock solutions (1,000 mg/L) for each compound were prepared in acetone and diluted into 50% sucrose solution (w/v) and stored at −20°C. The final concentration of 0.32 mg/L acetamiprid and 0.27 mg/L difenoconazole used for nursing honey bees’ exposure were diluted in sucrose solution from stock solutions (Kubik et al., 2000; Tong et al., 2018). All treatment solutions were freshly prepared daily.

### Honey bee rearing

For the laboratory experiment, three healthy colonies of honey bees (*A. cerana*) were obtained from outside the hives kept in the front of the building of the Environment and Plant Protection Institute, Chinese Academy of Tropical Agricultural Sciences, Haikou (N19°59′9'' and E110°19′30''), China. These colonies were maintained according to standard beekeeping practices, and they did not present visible symptoms of any known diseases. Prior to our study, these hives were not treated with any pesticides.

### Experimental design of laboratory exposures

Frames of late-stage capped brood from the three colonies were collected and transferred to an artificial climate incubator (34°C, 60% relative humidity; darkness) in the laboratory to monitor the emergence of *A. cerana* workers. Approximately 2000 newly emerged bees within a period of 12 h were marked on the thorax with a marker pen and returned to a single hive so that they could develop into nursing bees under the same conditions. After 7 days, these marked bees were captured, transferred to the laboratory, and distributed into iron cages (13 cm × 6 cm × 10 cm), and they were fed *ad libitum* with 50% sucrose solution (w/v) for acclimation 24 h before the beginning of the exposure experiment.

Next, the marked bees were divided into four treatment groups: control (C), ingestion of 50% sucrose solution free of pesticides; acetamiprid (TA), exposed to the 50% sucrose solution containing acetamiprid at the concentration of 0.32 mg/L; difenoconazole (TD), exposed to the 50% sucrose solution containing difenoconazole at the concentration of 0.27 mg/L; difenoconazole + acetamiprid (TDA), exposed simultaneously to the 50% sucrose solution containing the combined concentrations of 0.27 mg/L difenoconazole and 0.32 mg/L acetamiprid. Each experimental group was assayed in six replicates (six iron cages) and each cage contained 30 worker bees. The treatment solutions were replaced every 24 h. The exposure experiment lasted 10 days, and the dead bees in each group were recorded daily. The amount of solutions in the feeders was weighed daily before they were placed in the cages and again after they were removed from the cages. The difference was equivalent to the total amount of food consumed by live bees on the previous day, and then, we calculated the amount of food consumed by each honey bee. At the end of the test, the surviving honey bees were collected from each treatment and weighed, and then, their heads were removed by cutting with a blade and their whole guts were carefully collected by pulling the sting from the end of the abdomen using sterile forceps. These guts and heads were transferred into separate 1.5 mL centrifuge tubes and immediately frozen in liquid nitrogen and stored at −80°C until further analysis.

### DNA extraction and amplification

From each treatment group, 12 guts were pooled together as a biological sample, and three replicates were used (a total of 12 samples). Total genomic DNA from samples was extracted using the PowerSoil^®^ DNA Isolation Kit (MO BIO Laboratories) according to the manufacturer’s protocol. DNA quality and quantity were assessed by the ratios of 260 nm/280 nm and 260 nm/230 nm, respectively. Then, DNA was stored at −80°C until further processing. The V3–V4 region of the bacterial 16S rRNA gene was amplified with the universal primer pair (forward primer, 5'- ACT​CCT​ACG​GGA​GGC​AGC​A-3'; reverse primer, 5'- GGACTACHVGGGTWTCTAAT-3'), and the ITS region for fungi was amplified using barcoded primers (forward primer, 5'-CTT​GGT​CAT​TTA​GAG​GAA​GTA​A-3'; reverse primer, 5'-GCT​GCG​TTC​TTC​ATC​GAT​GC-3'). All PCR reactions were carried out with Phusion^®^ High-Fidelity PCR Master Mix (New England Biolabs). Illumina MiSeq sequencing and bioinformatics analyses were performed by a commercial company (Biotree, Shanghai, China).

### 16S rRNA gene sequence and ITS sequence analysis

Operational taxonomic unit (OTU) data were summarized by using Uparse software (Uparse v7.0.1001). Sequences with ≥97% similarity were assigned to the same OTUs. Bacterial species annotation was performed by the SILVA database, and fungi species annotation was performed by the UNITE database. Alpha and beta diversities were calculated using QIIME software. Principal coordinate analysis (PCoA) was performed on Bray–Curtis distance matrices. Analysis of similarity (ANOSIM) was performed to determine the differences among groups. Linear discriminant analysis (LDA) of Effect Size (LEfSe) was assessed with the LEfSe tool. Flora's relative abundance between samples was compared by MetaStat analysis.

### Gene expression analysis

The total RNA of three honey bee heads or three honey bee gut samples was pooled together as a biological sample and ground using a TissueLyser-64 instrument with a CoolPrep adapter. RNA was isolated following the manufacturer’s instructions using the Eastep^®^ Super Total RNA Extraction Kit (Promega, United States). Per each treatment, three biological replicates were isolated. 1,000 ng RNA was synthesized into cDNA using the GoScript Reverse Transcription system (Promega, A5001), according to the manufacturer’s protocol. Primer sequences were taken from the literature or self-designed using the NCBI primer-blast tool. Sequences of used primers are shown in Supplementary Table S1. For all performed analyses, *β-actin* was used as a housekeeping gene for normalization. Relative abundance of the mRNA level was assessed using a QuantStudio 6 Flex real-time qPCR System (Applied Biosystems, United States) by using an SYBR Green PCR kit (Aidlab, Beijing, China). The relative expression of the target genes was calculated using 2^−ΔΔCT^.

### Statistical analysis

Statistical calculations were performed using SPSS software 19.0 (IBM, United States). Student’s *t*-test was used for two-group comparisons. One-way ANOVA with an LSD test was used for four-group comparisons. The Kaplan–Meier survival curve and long-rank tests were used for survival analysis. The significance level used in all tests was *p* ≤ 0.05. Prism version 8.0 software (GraphPad, San Diego, CA, United States) was used to make the statistical figures.

## Results

### Acetamiprid and difenoconazole uptake and effects on honey bees’ survival and weight

**FIGURE 1:**
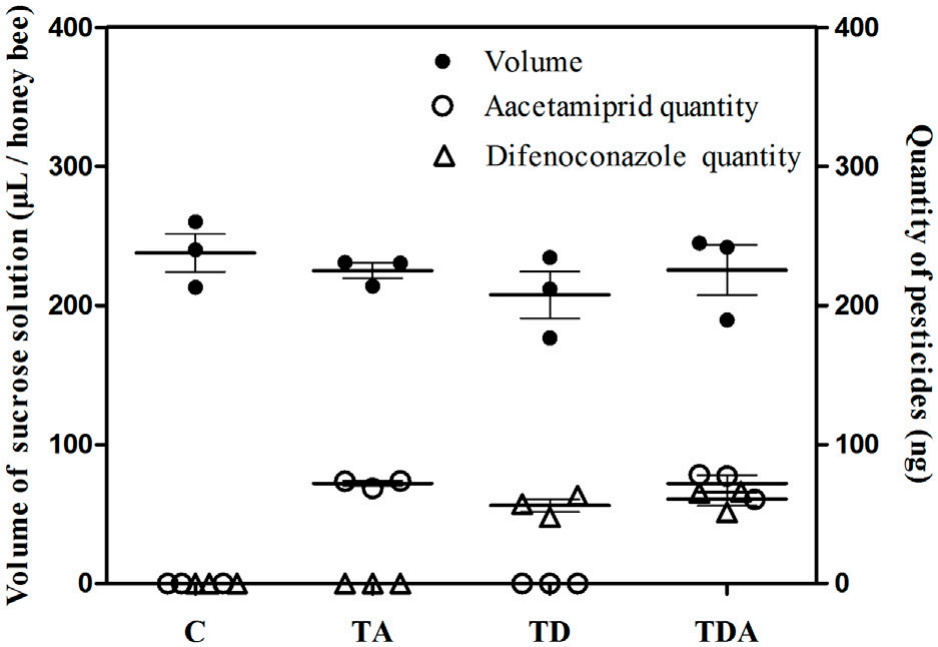
Mean uptakes of sucrose solution containing acetamiprid or/and difenoconazole and these two pesticides quantities per honey bee for 10 d feeding. The data (mean ± SEM) shown are representative of three colonies. Statistical analyses were performed using one-way ANOVA (volume) and Student’s *t*-test (quantity). C: control; TA: acetamiprid treated; TD: difenoconazole treated; and TDA: difenoconazole + acetamiprid treated. The following figures are the same.

The concentration of acetamiprid and difenoconazole, alone or in combination, in sucrose solution did not significantly influence the volume of solution taken up per honey bee during the 10 days of feeding (ANOVA; F = 0.743; d f = 3, 8; *p* = 0.556; Figure 1). The total doses of acetamiprid taken up per honey bee averaged 72.03 ± 1.78 and 72.11 ± 5.74 ng for TA- and TDA-treated groups, respectively, and there was no significant difference between the two groups (*t*-test, *t*_4_ = −0.015 and *p* = 0.989). Likewise, the total doses of difenoconazole consumed per honey bee averaged 56.05 ± 4.56 and 60.85 ± 4.84 ng for TD- and TDA-treated groups, respectively, and there was no significant difference between the two groups (*t*-test, *t*_4_ = −0.721 and *p* = 0.511).

**FIGURE 2:**
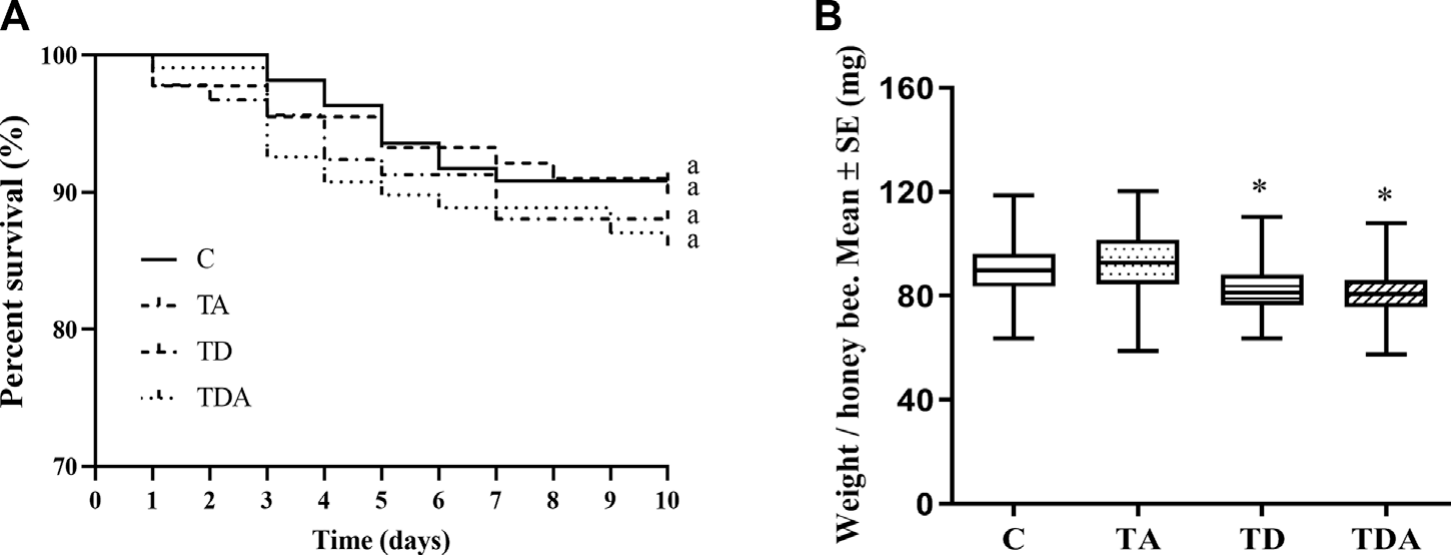
Effects of acetamiprid or/and difenoconazole 10 d on the honey bee workers’ survival **(A)** and body weight **(B)**. **(A)** The percent survival of workers after pesticide exposure is shown as a Kaplan–Meier survival curve. The same letters behind the curves indicate no significant differences between treatments (log-rank (Mantel–Cox) test: χ^2^ = 1.432; d f = 3; *p* = 0.698). **(B)** The data (mean ± SEM) shown are representative of three colonies. Statistical analyses were performed using one-way ANOVA. * indicates a significant difference compared to the control (*p* < 0.05).

After 10 days of exposure experiment, there was no significant difference in the percentage survival of honey bees among the four treatments (log-rank (Mantel–Cox) test: χ^2^ = 1.432; d f = 3; *p* = 0.698; Figure 2A). All the exposed honey bees behaved normally, and no honey bees that stopped moving were observed.

While the weights of honey bees were significantly reduced in TD- and TDA-treated groups when honey bee workers were exposed to an environmentally relevant concentration of acetamiprid and difenoconazole, alone or in combination for 10 days, there was no difference in the TA-treated group as compared with the control (F = 25.33; d f = 3, 334; *p* < 0.0001; Figure 2B).

### Effects of acetamiprid and difenoconazole on the honey bee gut microbiota

In order to obtain the honey bees’ gut microbes under natural conditions, these newly emerged honey bees were maintained in a single hive for 7 days because the newly emerged worker lacked gut microbes and gained large characteristic communities in the ileum and rectum within 4–6 days within hives. A total of 920,091 16S rRNA genes and 873,261 ITS clean reads were obtained from pyrosequencing 12 samples [(3 treatments +1 control) × 3 replicates], each sample comprising pooled DNA from 12 guts of honey bee (Supplementary Tables S2, S3). For 16S data, 821,985 effective reads with an average length of 423 bp (range: 420–426 bp) were retained after stringent quality filtering (Supplementary Table S2). A maximum of 48 unique operational taxonomic units (OTUs were clustered based on a 97% similarity cut-off). Among them, 41 OTUs have been found in all samples (Figure 4A). Similarly, for fungi, 869,213 effective reads with an average length of 234 bp (range: 193–289 bp) were retained after stringent quality checking (Supplementary Table S3). Based on the 97% similarity level, all of the effective reads were clustered into 800 OTUs. Among them, 525 OTUs were detected in all samples (Figure 6A). The curves of OTU rank and rarefaction were calculated (Supplementary Figure S1). Rarefaction for both the 16S (Supplementary Figures S1A, B) and ITS (Supplementary Figures S1C, D) data showed a plateau supporting the estimates of richness.

### Bacterial diversity and composition in response to acetamiprid and difenoconazole, alone and in combination

**FIGURE 3:**
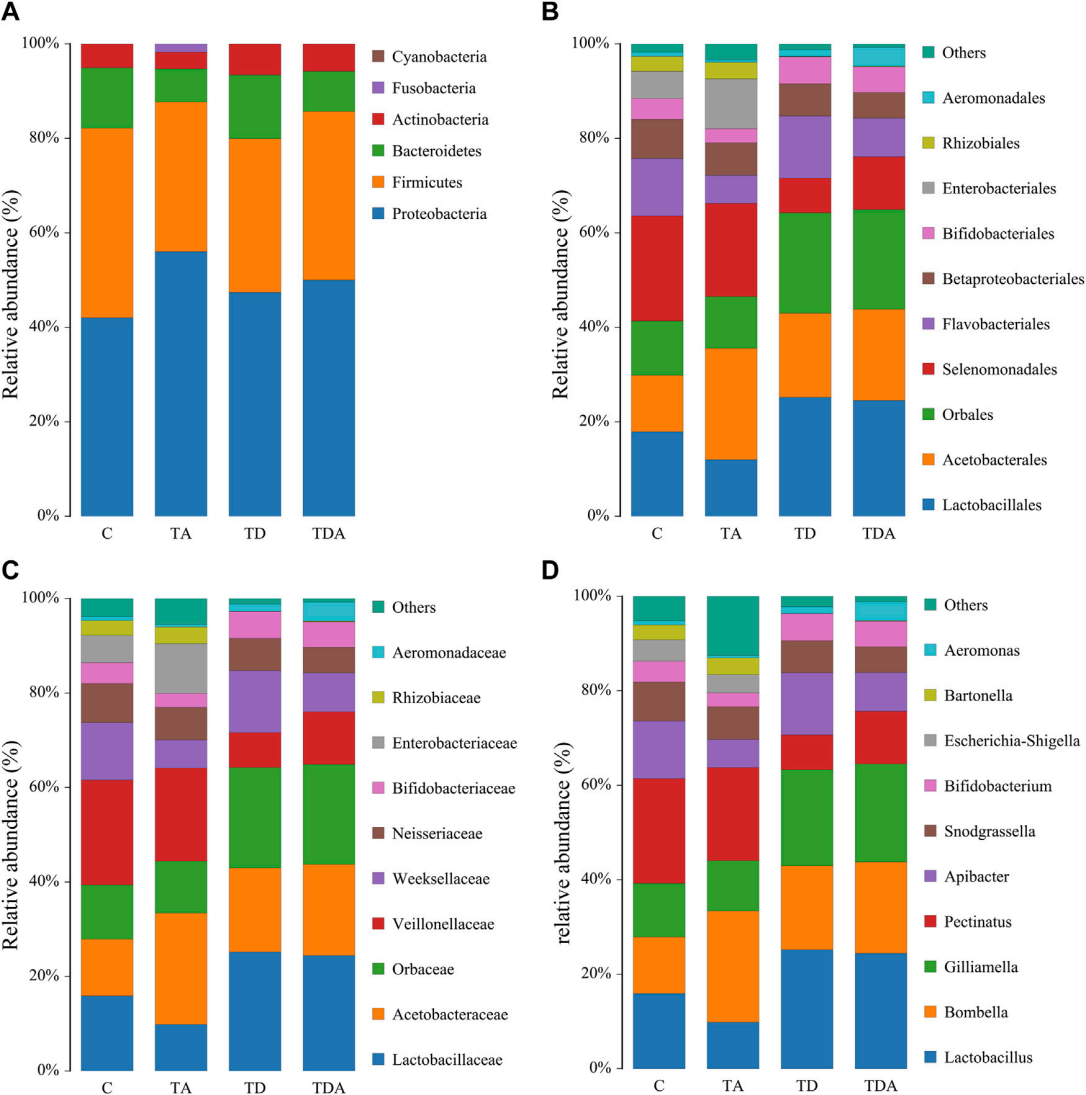
Relative abundance of the dominant gut bacterial communities in *Apis cerana* at the phylum **(A)**, order **(B)**, family **(C)**, and genus **(D)** levels. Each column represents the relative abundance of each bacterial taxon within a group.

The results of cluster analysis are that the gut bacterial communities belonged to 6 phyla, 9 classes, 15 orders, 16 families, 24 genera, and 31 species, as shown in Figure 3. At the phylum level, Proteobacteria (48.67%), Firmicutes (35.04%), Bacteroidetes (10.58%), and Actinobacteria (5.32%) were found to be the most abundant phyla (>1%). The family Lactobacillaceae (19.29%) of class Bacilli (20.28%), Acetobacteraceae (18.03%) of class ɑ-Proteobacteria (19.63%), and members of class γ-Proteobacteria (29.04%) were the most abundant community. The sequences from γ-Proteobacteria were predominantly dominated by members of the order Orbaceae (genera *Gilliamella*). Along with Orbaceae, reads assigned to Enterobacteriales (genera *Escherichia–Shigella*, *Serratia*, *Kosakonia*, *Klebsiella*, *Tatumella*, *Salmonella*, and *Enterobacter*), Aeromonadales (genera *Aeromonas*), and β-proteobacteriales (genera *Snodgrassella*) were also observed. In addition, members of Bacteroidia (10.58%) and Negativicutes (14.77%) were predominantly assigned to the family Weeksellaceae and Veillonellaceae, respectively.

**FIGURE 4:**
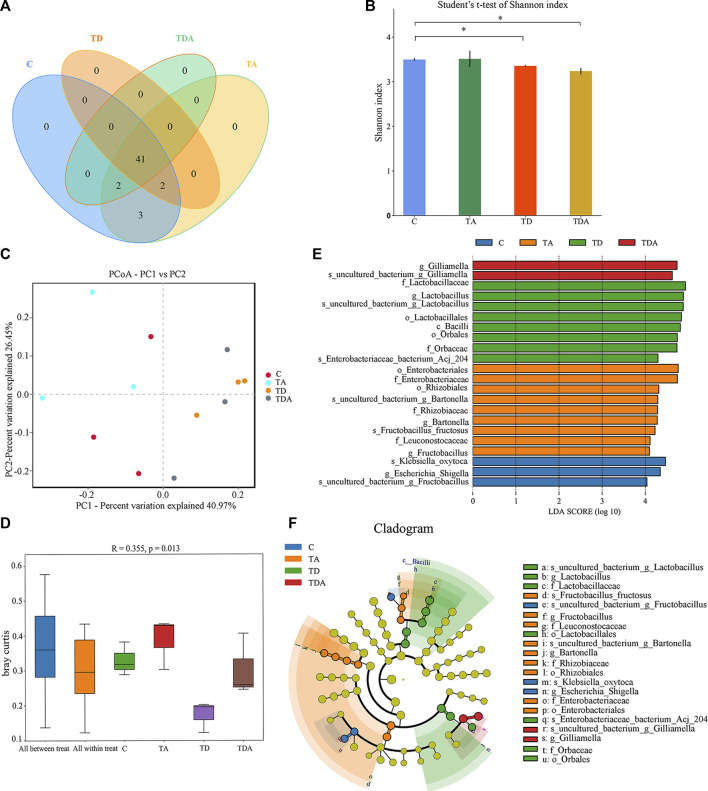
Gut bacterial microbiome alterations when *Apis cerana* exposed to acetamiprid or/and difenoconazole at environmentally relevant concentrations. **(A)** Venn diagram showing the numbers of unique and shared OTUs in the gut bacterial microbiota of *Apis cerana* between the pesticide-treated and the control groups. **(B)** Alpha diversity is measured by the Shannon index. Data (mean ± SEM) were analyzed by Student’s *t*-test (*p < 0.05). **(C)** Beta diversity as shown on a PCoA plot. **(D)** The ANOSIM analysis revealed significant differences in the structure (ANOSIM, R = 0.355, *p* = 0.013) of gut bacteria among these treatments. **(E)** LefSe (LDA >4 logs) analysis showing differentially abundant gut bacteria among samples with different haze levels. **(F)** Cladogram showing phylotype differences between pesticide-treated *versus* control honey bees.

Four alpha diversity parameters, namely, the Shannon, Simpson, ACE, and Chao1 indices, were selected for community diversity and richness comparisons. The alpha diversity of gut bacteria of the honey bee in TD and TDA groups had significant differences when compared with the control, as measured by the Shannon index (Figure 4B), while the Simpson index has no significance (Supplementary Figure S2A). In contrast to the diversity indices, there were no significant differences in the richness indices, as measured by the ACE and Chao1 indices (Supplementary Figures S2B, S2C).

The beta-diversity analysis was performed via a PCoA of Bray–Curtis distances to determine the similarity of bacterial communities. Results showed that the gut bacteria in the C and TA groups deviated from the TD and TDA groups (Figure 4C). The ANOSIM analysis revealed significant differences in the structure (ANOSIM, R = 0.355, *p* = 0.013) of gut bacteria among these treatments (Figure 4D). These data suggest that the gut bacterial community structures in honey bees were influenced by difenoconazole.

To identify the specific bacterial taxa associated with difenoconazole or/and acetamiprid exposure, we compared the gut bacteria of control and difenoconazole or/and acetamiprid treated honey bee using the linear discriminant analysis (LDA) Effect Size (LEfSe) and MetaStat method. LefSe analysis revealed that 3, 9, 8, and 2 bacterial taxa were enriched in C, TA, TD, and TDA groups, respectively (Figure 4E). The resulting cladogram showed that *Escherichia–Shigella* (genera), *Klebsiella oxytoca* (species), and *uncultured_bacterium_Fructobacillus* (species) were rich in control honey bees. The order Enterobacteriales and Rhizobiales, the family Enterobacteriaceae, Leuconostocaceae, and Rhizobiaceae, the genera *Bartonella* and *Fructobacillus*, and the species *uncultured_bacterium_Bartonella* and *Fructobacillus fructosus* were predominant in the TA group. The order Lactobacillales and Orbales, the family Lactobacillaceae and Orbaceae, the genera *Lactobacillus*, and the species *uncultured_bacterium_Lactobacillus* and *Enterobacteriaceae bacterium Acj 204* were predominant in the TD group, while *Gilliamella* (genera) and *uncultured_bacterium_Gilliamella* (species) were rich in the TDA group (Figure 4F). At the genus level, using MetaStat analysis, we found *Lactobacillus* and *Gilliamella* in the TDA group were increased, while *Klebsiella* decreased when compared with the control (*p* < 0.001, *p* = 0.027, and *p* = 0.045) (Supplementary Table S4). *Lactobacillus*, *Candidatus_Schmidhempelia,* and *Gilliamella* in the TD group were higher than in the control (*p* = 0.008, *p* = 0.021, and *p* = 0.038), while *Pectinatus* was lower than in the control (*p* = 0.047) (Supplementary Table S5). *Lactobacillus* and *Apibacter* in the TA group were lower than in the control (*p* = 0.007 and *p* = 0.049) (Supplementary Table S6).

### Fungal diversity and composition in response to acetamiprid and difenoconazole, alone and in combination

**FIGURE 5:**
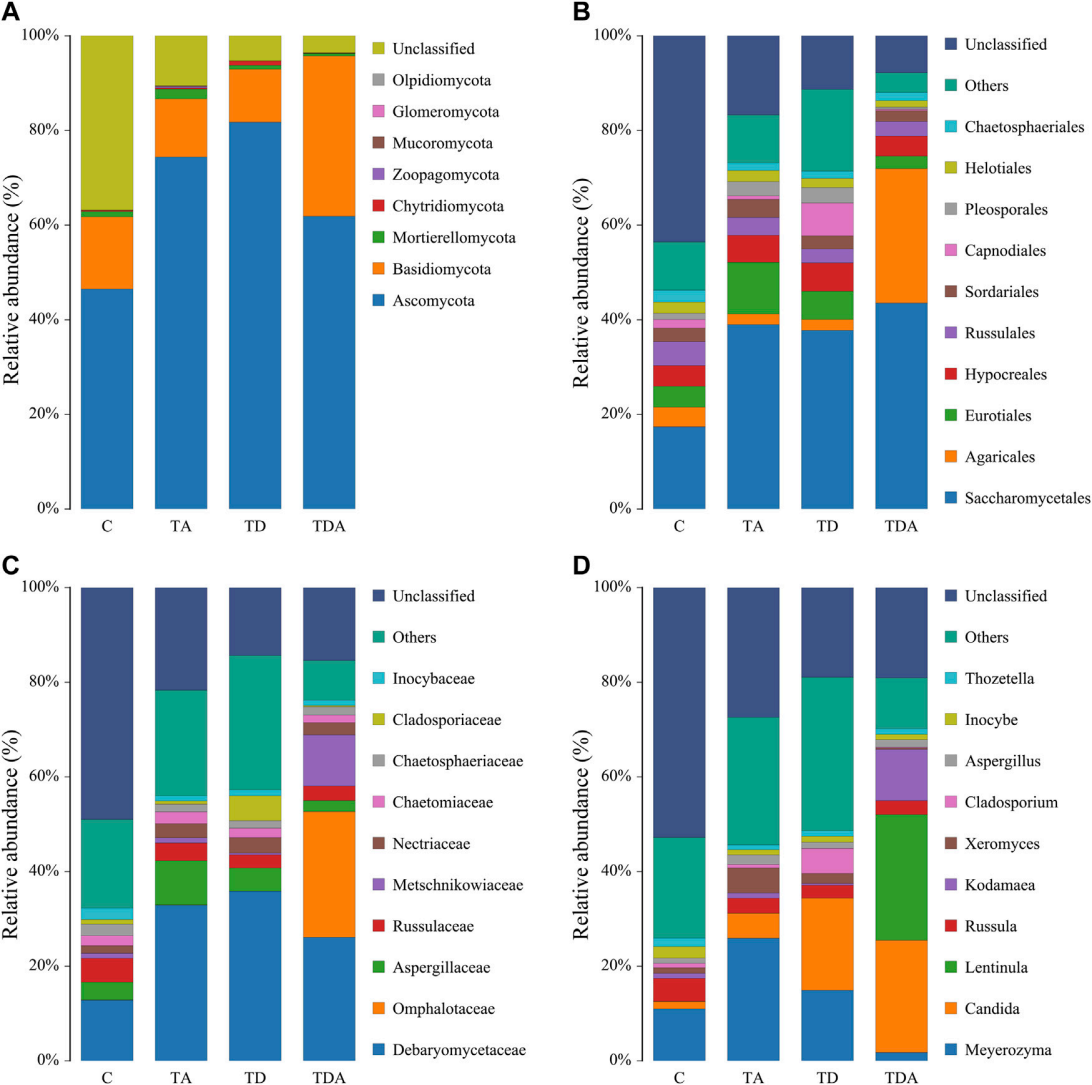
Relative abundance of the dominant gut fungal communities in *Apis cerana* at the phylum **(A)**, order **(B)**, family **(C)**, and genus **(D)** levels. Each column represents the relative abundance of each fungal taxon within a group.

The results of cluster analysis are that the gut fungal communities belonged to 8 phyla, 24 classes, 56 orders, 121 families, 188 genera, and 192 species, and the results do not contain unclassified (Figure 5). At the phylum level, Ascomycota (67.11%), Basidiomycotav (17.91%), and mortierellomycota (1.18%) were found to be the most abundant phyla (>1%). The classes with the highest abundance (>5%) were Saccharomycetes (34.98%), Sordariomycetes (14.86%), Agaricomycetes (15.16%), and Eurotiomycetes (7.85%). Saccharomycetales (34.98%), Agaricales (9.06%), Eurotiales (6.14%), and Hypocreales (5.14%) were the most abundant orders (>5%). The families with the highest abundance of fungi were Debaryomycetaceae (27.58%), Omphalotaceae (6.49%), Aspergillaceae (5.22%), and Russulaceae (3.62%). *Meyerozyma* (13.78%) and *Candida* (12.73%) of the family of Debaryomycetaceae were the most abundant genera.

**FIGURE 6:**
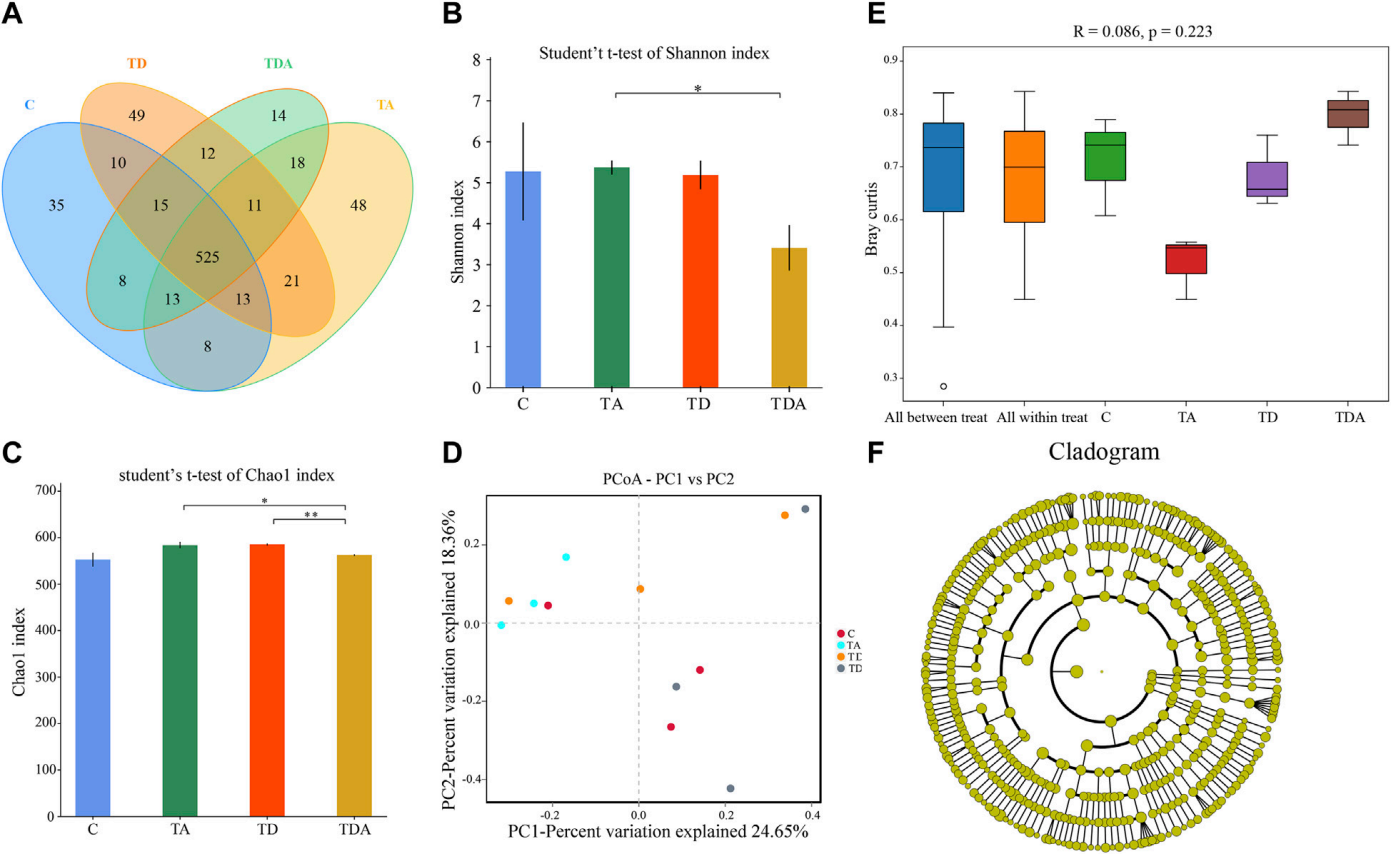
Gut fungal microbiome alterations when *Apis cerana* are exposed to acetamiprid or/and difenoconazole at environmentally relevant concentrations. **(A)** Venn diagram showing the numbers of unique and shared OTUs in the gut fungal microbiota of *Apis cerana* between the pesticide-treated and the control groups. **(B)** Alpha diversity is measured by the Shannon index. Data (mean ± SEM) were analyzed by Student’s *t*-test (*p < 0.05; **p < 0.01). **(C)** Beta diversity as shown on a PCoA plot. **(D)** The ANOSIM analysis revealed no significant differences in the structure (ANOSIM, R = 0.086, *p* = 0.223) of gut fungus among these treatments. **(E)** LefSe analysis revealed that no fungal taxa were enriched in the control and the different treatment groups.

The Shannon and Simpson indices showed that the fungal diversity had a significant difference between the TA and TDA groups (Figure 6B, Supplementary Figure S3A), the Chao1 and ACE indices showed that the fungal richness had a significant difference between the TD and TDA groups (Figure 6C, Supplementary Figure S3B); meanwhile, the Chao1 index also showed that the fungal richness had a significant difference between TA and TDA groups (Figure 6C), and there was no significant difference in all of the four alpha diversity indices when treated groups were compared with the control, but there may be a large variability among replicates in the control group. PCoA derived from the Bray–Curtis distance matrix of the fungal communities showed that samples from the control and the three treatment groups did not form separate clusters (Figure 6D). The ANOSIM analysis revealed that the acetamiprid or/and difenoconazole treatments did not have any significant impact on the fungal community structure (ANOSIM, R = 0.086, *p* = 0.223) (Figure 6E). LefSe analysis revealed that no fungal taxa were enriched in the control and the different treatment groups (Figure 6F). Using MetaStat analysis, we found two classes Saccharomycetes and Eurotiomycetes in the TA group were increased when compared with the control (*p* = 0.021 and *p* = 0.026) (Supplementary Table S7). Malasseziomycetes in the TDA group were decreased when compared with TA and TD groups (*p* = 0.005 and *p* = 0.039) (Supplementary Tables S8, S9). Leotiomycetes and Eurotiomycetes decreased, while Chytridiomycetes increased in the TDA group when compared with the TA group (*p* = 0.014, *p* = 0.024, and *p* = 0.010) (Supplementary Table S8), but Sordariomycetes and Dothideomycetes in the TDA group were decreased when compared with the TD group (*p* = 0.020 and *p* = 0.034) (Supplementary Table S9).

### Gene expression

**FIGURE 7:**
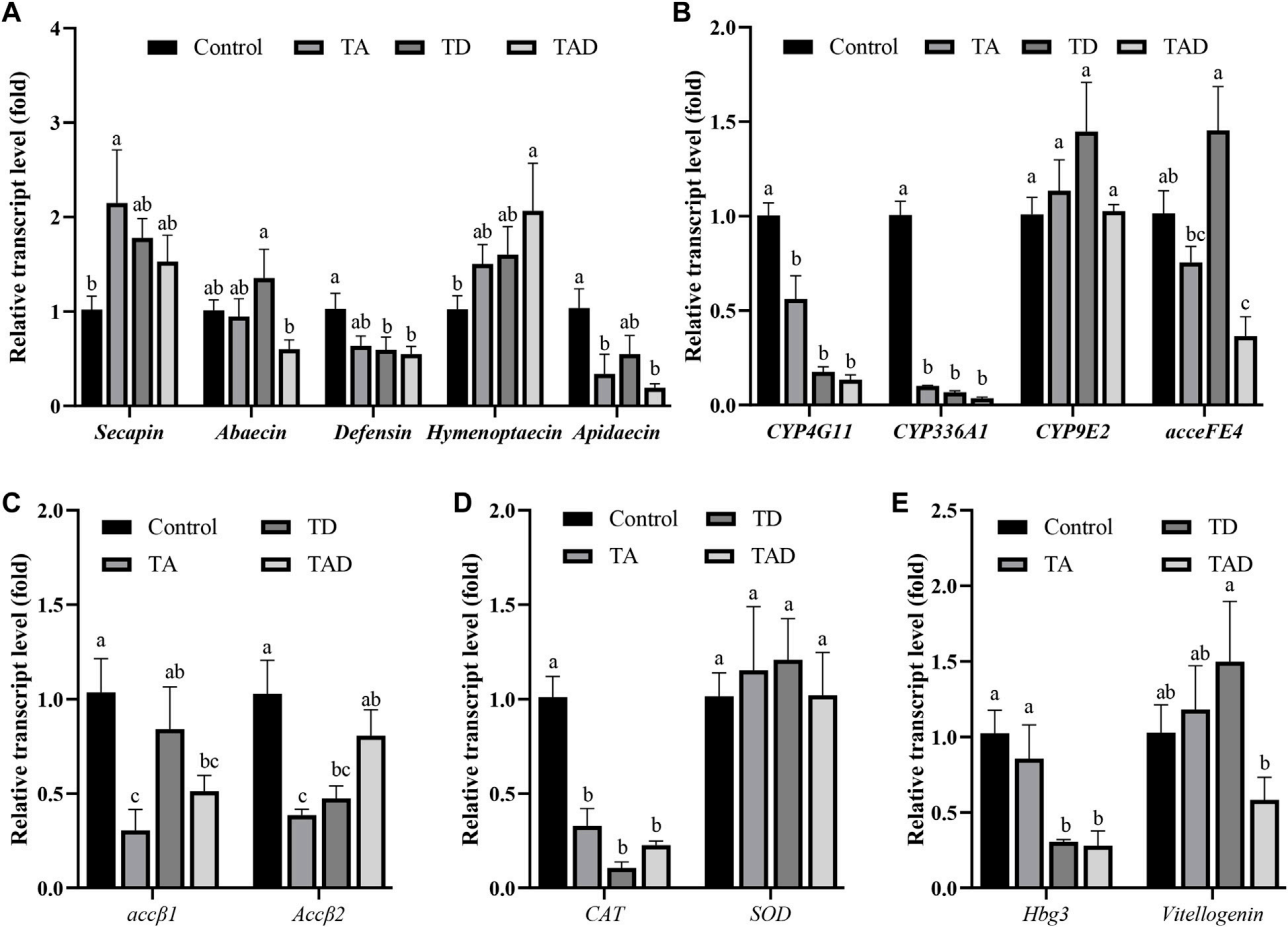
Normalized gene expression of immunity, *secapin*, *abaecin*, *defensin*, *hymenoptaecin*, and *apidaecin* **(A)**, detoxification, *CYP4G11*, *CYP9E2*, *CYP336A1*, and *acceFE4* **(B)**, acetylcholine receptor, *accβ1* and *accβ2* **(C)**, antioxidant reactions, *CAT* and *SOD* **(D)**, and hormonal regulation-related genes, *hbg-3* and *vitellogenin*, **(E)** in the head of *Apis cerana* exposed for 10 d to acetamiprid or/and difenoconazole at environmentally relevant concentrations. Data are means ± SEM. One-way ANOVA was performed for all treatments (LSD test), and bars topped with the same letters are not statistically different at *p* = 0.05.

**FIGURE 8:**
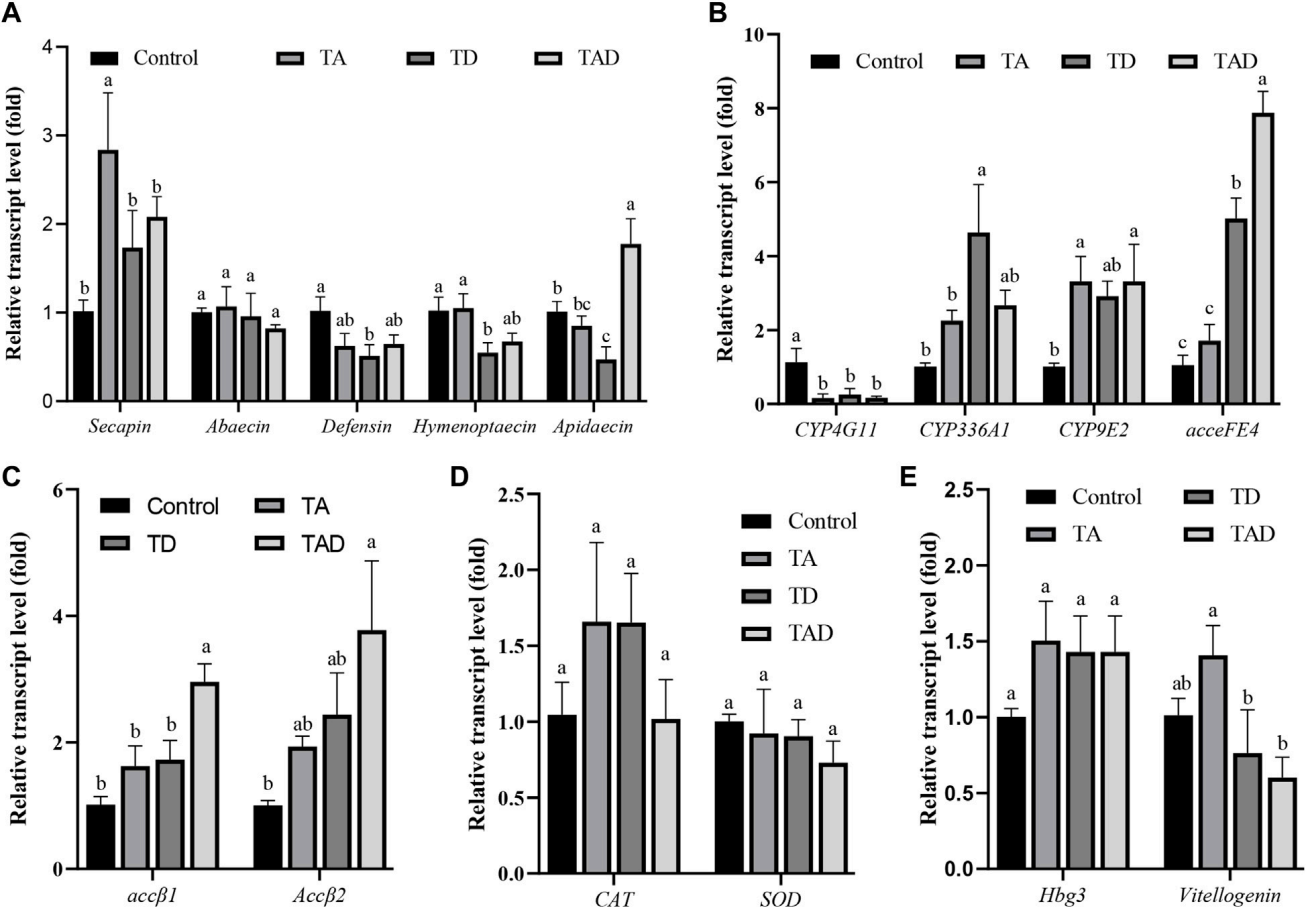
Normalized gene expression of immunity, *Secapin*, *abaecin*, *defensin*, *hymenoptaecin*, and *apidaecin* **(A)**, detoxification, *CYP4G11*, *CYP9E2*, *CYP336A1*, and *acceFE4* **(B)**, acetylcholine receptor, *accβ1* and *accβ2* **(C)**, antioxidant reactions, *CAT* and *SOD* **(D)**, and hormonal regulation-related genes, *hbg-3* and *vitellogenin*, **(E)** in the gut of *Apis cerana* exposed for 10 d to acetamiprid or/and difenoconazole at environmentally relevant concentrations. Data are means ± SEM. One-way ANOVA was performed for all treatments (LSD test), and bars topped with the same letters are not statistically different at *p* = 0.05.

To investigate the molecular effects of pesticide exposure, we assessed transcriptional alterations of selected genes (Figures 7, 8). These genes are involved in functions such as immunity, acetylcholine receptor, detoxification, antioxidant reactions, and hormonal regulation, which are activated in response to environmental stressors in honey bees.

### Transcriptional alteration of immunity-related genes

Compared to control honey bees, the expression of *secapin* transcripts was significantly upregulated in the TA group, while the expression levels of *defensin*, *hymenoptaecin,* and *abaecin* were not significantly affected, whether in the head (Figure 7A) or gut (Figure 8A) (*p* < 0.05). In addition, the expression of *apidaecin* was significantly downregulated in the head of TA honey bees, while in the gut, it was not altered. The expression of *defensin* was significantly suppressed in the head of the TD group, while *abaecin*, *hymenoptaecin,* and *apidaecin* transcripts were not evidently altered (*p* < 0.05). In addition, difenoconazole significantly inhibited the expression of *defensin*, *hymenoptaecin,* and *apidaecin* in the gut of honey bees (*p* < 0.05). When honey bees were co-exposed to acetamiprid and difenoconazole, the *hymenoptaecin* transcript was significantly upregulated in the head, while the *apidaecin* transcript was significantly downregulated (*p* < 0.05). In contrast, the expression of *apidaecin* in the gut was significantly upregulated (*p* < 0.05).

### Transcriptional alteration of detoxification-related genes

Significant differences were observed in the expression of detoxification genes between honey bees treated with pesticides and control honey bees (Figure 7B; Figure 8B). Acetamiprid and difenoconazole, alone or in combination, significantly decreased the expression levels of *CYP4G11* in the head and gut. It is worth noting that acetamiprid or difenoconazole, especially their mixture, showed the *CYP4G11* and *CYP336A1* transcripts with the strongest significant downregulation in the head (*p* < 0.01). A similar trend was observed in the expression of *acceFE4*. Acetamiprid and difenoconazole mixtures significantly reduced the transcript of *acceFE4* in the honey bee head but strongly upregulated its expression in the gut (*p* < 0.05). A single exposure to acetamiprid or difenoconazole had no significant effect on the expression of *acceFE4* in the honey bee head; however in the gut, acetamiprid did not change its expression, but difenoconazole significantly induced it. Acetamiprid or/and difenoconazole did not change the *CYP9E2* transcript in the honey bee head, but they extremely induced the *CYP9E2* transcript in the gut (*p* < 0.05).

### Transcriptional alteration of genes encoding acetylcholine receptors *Accβ1* and *Accβ2*

Acetamiprid significantly reduced the expression of *Accβ1* and *Accβ2* in the honey bee head (*p* < 0.05), while difenoconazole and acetamiprid–difenoconazole mixture led to only a weak suppression of *Accβ1* and *Accβ2* transcripts in the head (Figure 7C). In contrast, the acetamiprid and difenoconazole mixture significantly increased the expression of *Accβ1* and *Accβ2* in the honey bee gut, while single acetamiprid or difenoconazole led to only a weak induction, and the difference did not reach statistical significance (Figure 8C).

### Transcriptional alteration of antioxidant reaction-related genes

The expression of *CAT* in the honey bee head was significantly suppressed by acetamiprid or/and difenoconazole exposure (*p* < 0.05) (Figure 7D), while in the gut, the expression of *CAT* was not altered (Figure 8D). Exposure to acetamiprid or/and difenoconazole had no significant effects on the expression of *SOD*, whether in the head or gut (Figures 7D, 8D).

### Transcriptional alteration of hormonal regulation-related genes

Difenoconazole and acetamiprid–difenoconazole mixtures significantly downregulated the expression of *hbg-3* in the honey bee head (*p* < 0.05). In addition to this, the expression changes of *hbg-3* and *vitellogenin* in both the honey bee head and gut were observed when compared with the control, but the differences did not reach statistical significance (Figures 7E, 8E).

## Discussion

There is a concern about the potential health risks of bee populations throughout the world that are exposed to a cocktail of pesticides, including neonicotinoids and fungicides (David et al., 2015; Mitchell et al., 2017). The focus of concern and controversy is the dose of pesticides, and whether the bees are likely to be exposed to them in the field (David et al., 2016). Thus, obtaining more information about field-realistic pesticide exposure on the effects on honey bees’ health is vital to take this debate forward. In this study, we examined the effects of acetamiprid or/and difenoconazole on the survival, sucrose consumption, body weight, and gut microbiota composition of *A. cerana* at environmentally relevant concentrations and the molecular effects associated with them.

It is difficult to accurately estimate the concentrations of pesticides that honey bees encounter in the field. In this study, the doses (0.32 mg/kg acetamiprid and 0.27 mg/kg difenoconazole) administered to honey bees were detected from beebread, which is the main food for nurse workers. The total doses of acetamiprid uptakes in our experiment were 72.03 (alone) and 72.11 (combination) ng/honey bee for nursing honey bees. The dose corresponds to about 0.02 times the acetamiprid oral LD_50_ (3.208 μg/honey bee) obtained in a previous study for *A. cerana* (Han et al., 2017). The total doses of difenoconazole uptakes in our experiment were 56.05 (alone) and 60.85 (combination) ng/honey bee for nursing honey bees. These doses were far below those detected from the honey sample (1 μg/kg) (Herrera López et al., 2016) and much lower than the dose used by Iverson et al. (2019) (200 ng/honey bee). Considering the pesticide degradation in the environment and the honey bee feed on a mix of contaminated and uncontaminated plant pollen or nectar, we assume that honey bees that received these low doses conform to the agricultural field’s actual situation.

From the data presented, it has been indicated that acetamiprid or/and difenoconazole continuous exposure almost did not affect survival and food consumption, but difenoconazole decreased the weight of honey bees. Our previous study showed that the total mean doses of acetamiprid were 198.8 (alone) and 109.8 (combination) ng/honey bee for newly emerged bees and 1.39 (alone) and 1.36 (combination) μg/honey bee for forager bees, which severely affected *A. cernan* survival (Han et al., 2019). From these experimental results, we can infer that there was a dose-dependent effect of acetamiprid on honey bees’ survival. In addition, one of the main reasons for the differences may be honey bees’ age differences. Difenoconazole affected the weight of honey bees because this fungicide may be accumulated in honey bee tissues; honey bees need to increase more of their energetic investment in detoxification and immunity and promote the growth of beneficial microbiota.

Honey bees acquire their microbiota after emergence through interactions with their hive environment and social exchange (Guo et al., 2015). Our results demonstrated that the gut bacterial communities of *A. cerana* adult workers comprise four major phyla, Proteobacteria, Firmicutes, Bacteroidetes, and Actinobacteria. This result is consistent with Luo et al.’s (2020) reports. At the genus level, previous studies showed that *Lactobacillus*, *Snodgrassella*, and *Gilliamella* were the major genera (Guo et al., 2015; Huang et al., 2018; Luo et al., 2020). In addition to this, we found *Bombella*, *Pectinatus,* and *Apibacter* also have very high abundance in our samples. Other genera of *Aeromonas*, *Atopobium*, *Bartonella*, *Bifidobacterium*, *Dysgonomonas*, *Enterobacter*, *Candidatus_Schmidhempelia*, *Escherichia–Shigella*, *Fructobacillus*, *Klebsiella*, *Kosakonia*, *Pseudomonas*, *Salmonella*, *Sebaldella*, *Serratia*, *Snodgrassella*, and *Tatumella* were observed. The occurrence of these genera in the gut of *A. cerana* and the exact role played by them will be investigated in future studies.

There is accumulating evidence indicating that gut microbiota is critical in the maintenance of physiological homeostasis, and perturbing it can induce detrimental effects (Sun et al., 2020; Xiong et al., 2020). Exposure to pesticides can influence the honey bee gut microbiota composition (Kakumanu et al., 2016; Motta et al., 2018; Liu et al., 2020). Our results showed that difenoconazole and the mixture of difenoconazole–acetamiprid significantly shaped the composition of the gut bacteria of *A. cerana* adult workers, but acetamiprid did not impact the gut bacterial community.

Agrochemical substances can result in microbial dysbiosis, with diversity and composition modifications that may increase a bee’s vulnerability to other abiotic and biotic stressors (Muñoz-Colmenero et al., 2020). In this study, the bacterial diversity was significantly reduced, but the richness was not affected under difenoconazole exposure (TD and TDA groups). It is commonly considered that high microbiota diversity has a positive impact on the host’s health due to more diverse microbial communities that are assumed to be more resistant and resilient to perturbations and external stressors (Shade et al., 2012), but the opposite argument is that higher richness of the core bacteria has positive effects on the bees’ health, while a higher diversity in non-core taxa is considered deleterious (Ribière et al., 2019). In this study, difenoconazole-treated honey bees have lower diversity, but the core strain of *Lactobacillus* and *Gilliamella* was significantly higher than in the control. *Lactobacillus* and *Gilliamella* bacteria are important members of the intestinal tract of honey bees (Kwong and Moran, 2016). *Lactobacillus* can produce antibacterial and antiviral compounds, such as organic acids, diacetyl, benzoate, and bacteriocins (Luo et al., 2020). *Gilliamella* can metabolize a diverse array of plant-produced carbohydrates and utilize glucose, fructose, mannose, and so on (Zheng et al., 2016). *Lactobacillus* and *Gilliamella* play an important role in resisting pathogenic bacteria and the immune protection of honey bees. Our results also found that *Pectinatus* abundance is relatively reduced in response to difenoconazole exposure. *Pectinatus* is a recurrent brewing spoilage bacterium (Rodríguez-Saavedra et al., 2021). This bacterium in the honey bees’ gut may come from the environment. There is little scientific information concerning the functional roles of this bacterium in the honey bees’ gut. In addition, it is well known that *Escherichia–Shigella* is a known opportunistic pathogen (Zhou et al., 2018). In this study, the abundance of *Escherichia–Shigella* changed in TD and TDA groups, but this did not reach significance from a statistical standpoint. It is worth mentioning that the *Aeromonas* strains and *Aeromonas veronii* were much more abundant in those honey bees of the TDA group. *Aeromonas veronii* is a Gram-negative bacteria, which is a widely distributed novel pathogen that can affect humans and animals (Huang et al., 2020). Difenoconazole, as a fungicide, has bactericidal properties. Honey bees’ recurrent consumption of food containing this fungicide may have caused the death of sensitive bacteria, thus disturbing gut physiological homeostasis. So, further research is needed to isolate these bacteria in honey bees’ guts and determine their sensitivity to difenoconazole.

Furthermore, in the current study, the alpha-diversity and beta-diversity analysis indicated that acetamiprid does not significantly affect the gut bacteria composition of honey bees. PCoA also showed that the gut community compositions of exposed and control honey bees were similar between the two groups. Our result was similar to Liu et al. (2020), who reported thiacloprid (acetamiprid and thiacloprid both belongs to cyano-substituted neonicotinoids) exposure did not impact the abundance of honey bee gut microbiome in the low dose (0.2 mg/L), but significantly reduced abundance in the high dose (0.6 and 2.0 mg/L). One possible explanation as to why low-dose acetamiprid does not impact the honey bee gut microbiome could be because once acetamiprid entered the midgut (the primary place of metabolism), it was quickly eliminated from or metabolized by honey bee cells before it reaches the hindgut (the primary place of bacteria colonization). Although low-dose acetamiprid did not affect the gut bacteria composition, the core strain of *Lactobacillus* and *Apibacter* was significantly lower than in the control. *Lactobacillus* spp. are important probiotics, and they can secrete bacteriostatic substances (bacteriocins and lactic acid) to protect their hosts (Tang et al., 2021). *Apibacter* is prevalent in the gut of *A. cerana*; it may provide hosts with vital benefits (Zhang et al., 2021). The decrease in the abundance of *Lactobacillus* and *Apibacter* may lead to poor health of honey bees.

Similar to bacteria, fungi also play an important role in the maintenance of intestinal homeostasis, although fungi accounted for a small proportion microbiota of the alimentary canals of the bees (Khan et al., 2020). Our results showed that the fungal communities of honey bee gut microbiota were dominated by members of *Ascomycota* and *Basidiomycota*, consistent with Kakumanu et al. (2016) and Paris et al.’s (2020) previous reports. Alpha diversity analysis showed that chronic exposure to difenoconazole or/and acetamiprid had no effect on the fungal community structure when compared to the control group. Of note, although the overall beta diversity does not differ remarkably among pesticide treatments, we found a significantly lower Chao1 index (a marker of species richness) in honey bees with co-exposure to difenoconazole and acetamiprid *versus* single exposure. The results suggest that the cocktail of pesticides may exacerbate the disturbance of the gut microbiota of honey bees. In addition, at the genus level, we observed 188 genera; among them, *Meyerozyma*, *Candida*, and *Aspergillus* are the main central taxa. It is reported that these fungi correlate with several bacterial taxa including *Firmicutes*, *Bacteroides*, and *Faecalibacterium* (Nagpal et al., 2020). It is worth mentioning here that the gut fungi that truly colonized the gut of these subjects *versus* transient fungi that came through diets remain unknown. The genus *Candida* comprises various opportunistic species implicated in various gut-related diseases in humans (Nagpal et al., 2020). At present, the fungal flora remains largely unexplored in honey bees’ health, and this field needs further research.

It has been shown that gut microbiota composition correlates with altered gene expression in host tissues (Du et al., 2020). A previous study has shown that pesticides impacted the expression of immunity-related genes in honey bees’ guts (Aufauvre et al., 2014). In the current study, we found acetamiprid induced the expression of *secapin*, which is an antimicrobial peptide with activity against bacteria and fungi in the innate immune response (Lee et al., 2016). Additionally, we also found the expression of *defensin*, *hymenoptaecin,* and *apidaecin* was significantly downregulated in the gut of honey bees following chronic exposure to difenoconazole. Acetamiprid had no effect on the expression of these four genes related to immunity in the honey bee gut. *Defensin*, *hymenoptaecin,* and *apidaecin* are key antimicrobial components in insect innate immunity against invading pathogens. The immune system of honey bees consists of three pathways, the TLR, Imd, and Jak/STAT pathways. Each pathway displays different functions (Christen and Fent, 2017). Therefore, acetamiprid and difenoconazole might have different models of action on honey bees’ immune systems, which may lead to different compositions of gut microbiota.

Honey bees have detoxification systems that function in the metabolism of endogenous compounds and xenobiotics such as pesticides and plant toxins. It is well known that pesticides can change the expressions of detoxification-related genes in honey bees (Mao et al., 2017; Wu et al., 2017; Liu et al., 2021). In addition, honey bee gut dysbiosis leads to a change in P450 gene expression (Schwarz et al., 2016). The present study shows that the expression of P450 genes (*CYP4G11*, *CYP336A1*, and *CYP9E2*) and esterase gene (*acceFE4*) in the tissue of honey bee workers of *A. cerana* was altered after chronic exposure to acetamiprid or/and difenoconazole. *CYP4G11* belongs to the microsomal CYP4 family, which is involved in chemoreception, and its transcriptional alteration may induce chaotic behavior among honey bees (Mao et al., 2015; Wu et al., 2017). *CYP336A1* belongs to the microsomal CYP3 family, which plays an important role in protecting cells against oxidative damage (Zhu et al., 2016). *CYP9E2* belongs to the microsomal CYP9 family, which is involved in xenobiotic detoxification (Claudianos et al., 2006). In the head, we observed that the expression of *CYP4G11* and *CYP336A1* was significantly decreased in all pesticide treatment groups, and in the gut, the expression of *CYP4G11* was suppressed, while *CYP336A1* and *CYP9E2* were induced. Esterase *acceFE4* belongs to carboxylesterases, which are involved in xenobiotic metabolism (Ma et al., 2018). For the expression of *acceFE4*, the acetamiprid–difenoconazole mixture caused a significant decrease in the head and a significant increase in the gut. In the meanwhile, honey bees exposed to acetamiprid alone had no effect on *acceFE4* expression. These results demonstrated that low doses of acetamiprid and difenoconazole, especially the cocktail, seriously interrupted the detoxification gene expression in honey bees and enhances the pesticide risks for honey bees.

Nicotinic acetylcholine receptors (nAChRs) mediate fast cholinergic synaptic transmission in the insect nervous system and are important targets for insecticides. Alterations in neuronal signaling can have pronounced effects; for example, *A. mellifera* exposed to 3.8 ng/bee thiamethoxam caused locomotor deficits (Charreton et al., 2015). In this study, we found that acetamiprid triggered the downregulation of nAChR transcripts in the head, and the mixture of acetamiprid and difenoconazole triggered the upregulation of nAChR transcripts in the gut. Upregulation of nAChRs may represent a compensation reaction to the functional loss of the neuronal signaling upon exposure to neurotoxic pesticides (Christen et al., 2016).

In the case of the oxidative stress-related gene *CAT*, acetamiprid and difenoconazole significantly decreased their expression in the head, alone and in combination. *CAT* was involved in antioxidant reactions and xenobiotic detoxification (Aufauvre et al., 2014). In addition, we observed that difenoconazole and acetamiprid–difenoconazole mixtures led to the expressional downregulation of *hbg-3* in honey bees’ heads. The gene product of *hbg-3* is involved in the transition of nurse bees to foragers. In foragers, the hypopharyngeal glands are shrinking, and at the same time, the expression of *hbg-3* is increasing (Christen et al., 2019). From the results, we can infer that difenoconazole may prolong the development of nurse bees to foragers by downregulating *hbg-3* in the heads of honey bees. At present, *vitellogenin* has become widely accepted as a marker of honey bees’ overall health. It is an important regulator of life-span and foraging behavior, and changes in expression may have significant physiological effects (Christen et al., 2019). In the present study, acetamiprid and difenoconazole had no particularly pronounced effect on the expression of *vitellogenin* transcripts; thus, we think the transcripts showed only weak significance.

In conclusion, our results showed that acetamiprid or/and difenoconazole continuous exposure at concentrations that mimic environmental contamination almost did not affect the survival and food consumption of *A. cerana* under laboratory conditions. However, difenoconazole or acetamiprid–difenoconazole mixture-treated honey bees had structurally different bacterial communities compared to non-exposed colonies, but acetamiprid does not impact the gut bacterial community. Meanwhile, we also observed that acetamiprid or/and difenoconazole significantly altered the expression of genes linked to detoxification in the honey bee tissues. Furthermore, it is worth mentioning that the toxic effects of acetamiprid and difenoconazole co-exposure on the molecular level were greater than those of the single exposure. However, there were still several limitations in our study. Future experiments should be designed to observe the toxicological effects of pesticide cocktail on honey bee gut microbiomes under a real exposure scenario (field condition), and the molecular mechanism of the toxic effect should be elucidated with the methods of multi-omics, so as to find new targets for protecting honey bees. Ultimately, our study provides a good reference for farmers to know the toxic effects of pesticides on honey bees and how to select the chemical mixture if they produce synergistic interactions at environmentally realistic concentrations.

## Data Availability

Publicly available datasets were analyzed in this study. The accession number is PRJNA956719. This data can be found here: https://www.ncbi.nlm.nih.gov/bioproject/PRJNA956719.
