# Supplementary material for: Gut microbiota composition and gene expression changes induced in the Apis cerana exposed to acetamiprid and difenoconazole at environmentally realistic concentrations alone or combined
Source: Front Physiol. 2023 May 3;14:1174236. doi: 10.3389/fphys.2023.1174236 (PMC10226273; doi:10.3389/fphys.2023.1174236)
Supplement: Supplementary file 1 [file DataSheet1.docx]

**Supplementary material**

**Gut microbiota composition and gene expression changes induced in the *Apis cerana* exposed to acetamiprid and difenoconazole at environmentally realistic concentrations alone or combined**

Wensu Han ^1, 2*^, Zheyuan Ye ^1^, Yifan Gu ^1, 3, 4^, Yihai Zhong ^1, 2^, Jinglin Gao ^1, 2*^, Shan Zhao ^1, 2^, Shijie Wang ^1, 2^

^1^ Environment and Plant Protection Institute, Chinese Academy of Tropical Agricultural Sciences, Haikou, China

^2^ Bee Industry Technology Research Center, Chinese Academy of Tropical Agricultural Sciences, Haikou, China

^3^ Sanya Institute of China Agricultural University, Sanya, China

^4^ Department of Entomology, College of Plant Protection, China Agricultural University, Beijing, China

* Corresponding authors:

Wensu Han, E-mail: [hwswill8@126.com](mailto:hwswill8@126.com)

Jinglin Gao, E-mail: jinglin.g@163.com

**Table S1** Sequences of primer pairs used in the real-time quantitative PCR reactions

| Target gene | Primer sequence (5' - 3') | Gene classification | Source/Reference |
| --- | --- | --- | --- |
| *Secapin* | F: TTCGACCCGCCAACTTGGATTTAC | Antimicrobial peptide | Self-designed |
|  | R: TGGGAGGACATCTGACAGGTACTG |  |  |
| *abaecin* | F: ATCTTCGCACTACTCGCCAC | Antimicrobial peptide | Wu et al., 2020 |
|  | R: CCTGACCAGGAAACGTTGGA |  |  |
| *defensin1* | F: AGCCACTTGAGCATCCTGAG | Antimicrobial peptide | Wu et al., 2020 |
|  | R: CCGTTCTTGCAATGACCTCC |  |  |
| *hymenoptaecin* | F: CGTGTTGGTTGTCTTCTGCG | Antimicrobial peptide | Wu et al., 2020 |
|  | R: CACCATAGGCATCTCCCGTC |  |  |
| *apidaecin* | F: CTCCTCAACCTGCGCGTATTCG | Antimicrobial peptide | Self-designed |
|  | R: CGGTTTAGCTTCACGGCGTAGAC |  |  |
| *Accβ1* | F: CCTATGCGTGCTCGTGTTCTACC | Acetylcholine receptor | Self-designed |
|  | R: GGCAGGATCTTGCTGACCAGTAAC |  |  |
| *Accβ2* | F: AGCGTAACAAGTGTCGGCATCG | Acetylcholine receptor | Self-designed |
|  | R: CCGTAAGGCCACCAAGTGTGATC |  |  |
| *CYP4G11* | F: CGCAAAGAGAATGGGAAGG | Detoxifying enzyme  Cytochrome P450 | Shi et al., 2013 |
|  | R: CTTTTGTGTGACGGAGGTGC |  |  |
| *CYP9E2* | F: CTTTTTGCAGCCGTGAACGA | Detoxifying enzyme  Cytochrome P450 | Jiang et al., 2016 |
|  | R: AATCTGTCGTGCTTCTGGGG |  |  |
| *CYP336A1* | F: TGTTCGGTTATTTGCCATTCC | Detoxifying enzyme  Cytochrome P450 | Zhu et al., 2016 |
|  | R: GGTCTGCCAGTAAACTCTTCC |  |  |
| *AcceFE4* | F: CATGGTGGCGCATTTGTAGTAGGC | Detoxifying enzyme  carboxylesterase | Ma et al., 2018 |
|  | R: GGCCCAAGAACATCGTATCAATCC |  |  |
| *CAT* | F: GTCTTGGCCCGAACAATTTG | Antioxidant enzyme  catalase | Chao et al., 2019 |
|  | R: CATTCTCTAGGCCCACCAAA |  |  |
| *SOD2* | F: TTGCCATTCAAGGTTCTGGTT | Antioxidant enzyme  Superoxide dismutase | Chao et al., 2019 |
|  | R: GCATGTTCCCAAACATCAATACC |  |  |
| *hbg-3* | F: CAGTTTCTGCTGGATTTTCCTC | Hormone system | Self-designed |
|  | R: CGAGTTCTTGTCCTTCTTTTCA |  |  |
| *vitellogenin* | F: ACCAACGACTTCATGGGACC | Immune marker | Self-designed |
|  | R: CGCTGTCGCTGATCACATTG |  |  |
| *β-Action* | F: GTTTTCCCATCTATCGTCGG | Housekeeping gene | Self-designed |
|  | R: TTTTCTCCATATCATCCCAG |  |  |

**Table S2** Statistics of sample sequencing data processing result of gut bacterial microbiota

| Sample ID | Raw Reads | Clean Reads | Effective Reads | AvgLen (bp) | GC (%) | Q20 (%) | Q30 (%) | Effective (%) |
| --- | --- | --- | --- | --- | --- | --- | --- | --- |
| C1 | 79813 | 78895 | 67121 | 426 | 52.84 | 97.25 | 94.64 | 84.1 |
| C2 | 80032 | 79295 | 69400 | 422 | 52.03 | 97.43 | 95.02 | 86.72 |
| C3 | 80083 | 79275 | 70597 | 425 | 52.21 | 97.41 | 94.91 | 88.15 |
| TA1 | 80013 | 79054 | 70692 | 422 | 52.86 | 97.3 | 94.74 | 88.35 |
| TA2 | 61034 | 60254 | 51267 | 423 | 53.37 | 97.31 | 94.74 | 84 |
| TA3 | 72420 | 71685 | 61175 | 420 | 52.72 | 97.49 | 95.1 | 84.47 |
| TD1 | 80136 | 79216 | 72954 | 423 | 51.91 | 97.35 | 94.83 | 91.04 |
| TD2 | 80113 | 79220 | 72591 | 424 | 52.11 | 97.34 | 94.81 | 90.61 |
| TD3 | 79940 | 79055 | 72405 | 423 | 51.48 | 97.35 | 94.84 | 90.57 |
| TDA1 | 79893 | 79148 | 71959 | 423 | 52 | 97.47 | 95.05 | 90.07 |
| TDA2 | 80113 | 79220 | 72591 | 424 | 52.11 | 97.34 | 94.81 | 90.61 |
| TDA3 | 79940 | 79055 | 72405 | 423 | 51.48 | 97.35 | 94.84 | 90.57 |
| Total | 930302 | 920091 | 821985 | — | — | — | — | — |

**Table S3** Statistics of sample sequencing data processing result of gut fungal microbiota

| Sample ID | Raw Reads | Clean Reads | Effective Reads | AvgLen (bp) | GC (%) | Q20 (%) | Q30 (%) | Effective (%) |
| --- | --- | --- | --- | --- | --- | --- | --- | --- |
| C1 | 80274 | 77365 | 76904 | 237 | 46.05 | 99.76 | 99.27 | 95.8 |
| C2 | 43218 | 40769 | 40234 | 243 | 49.7 | 99.67 | 99.02 | 93.1 |
| C3 | 80461 | 79201 | 79017 | 226 | 37.71 | 99.85 | 99.47 | 98.21 |
| TA1 | 80137 | 77528 | 77223 | 241 | 47.13 | 99.78 | 99.32 | 96.36 |
| TA2 | 80086 | 77275 | 76571 | 233 | 46.07 | 99.79 | 99.36 | 95.61 |
| TA3 | 79839 | 77043 | 76802 | 244 | 45.89 | 99.79 | 99.37 | 96.2 |
| TD1 | 79855 | 77368 | 76952 | 244 | 48.65 | 99.76 | 99.28 | 96.36 |
| TD2 | 79921 | 76369 | 76102 | 242 | 46.46 | 99.79 | 99.37 | 95.22 |
| TD3 | 80052 | 76450 | 76282 | 210 | 47.67 | 99.84 | 99.47 | 95.29 |
| TDA1 | 80014 | 78482 | 78263 | 289 | 41.69 | 99.43 | 98.64 | 97.81 |
| TDA2 | 79950 | 72050 | 71853 | 204 | 47.26 | 99.8 | 99.37 | 89.87 |
| TDA3 | 65741 | 63361 | 63010 | 193 | 42.01 | 99.77 | 99.27 | 95.85 |
| Total | 909548 | 873261 | 869213 | — | — | — | — | — |

**Table S4** Metastats statistical result at genus taxonomy level of gut bacterial microbiota

| genus | Mean  (C) | Variance  (C) | Std.err  (C) | Mean  (TDA) | Variance  (TDA) | Std.err  (TDA) | P value | Q value |
| --- | --- | --- | --- | --- | --- | --- | --- | --- |
| Lactobacillus | 1.59E-01 | 5.34E-05 | 4.22E-03 | 2.45E-01 | 4.51E-04 | 1.23E-02 | ﹤0.001 | ﹤0.001 |
| Gilliamella | 1.13E-01 | 2.45E-03 | 2.86E-02 | 2.08E-01 | 1.30E-06 | 6.57E-04 | 0.0267 | 0.277 |
| Klebsiella | 6.33E-03 | 2.12E-05 | 2.66E-03 | 1.25E-04 | 1.71E-09 | 2.39E-05 | 0.0436 | 0.277 |
| Snodgrassella | 8.25E-02 | 1.97E-04 | 8.10E-03 | 5.41E-02 | 3.69E-04 | 1.11E-02 | 0.0527 | 0.277 |
| Fructobacillus | 2.01E-02 | 2.72E-04 | 9.53E-03 | 5.84E-04 | 8.26E-07 | 5.25E-04 | 0.0576 | 0.277 |
| Escherichia-Shigella | 4.59E-02 | 2.51E-03 | 2.89E-02 | 1.60E-04 | 2.22E-08 | 8.60E-05 | 0.129 | 0.399 |
| Atopobium | 6.52E-03 | 2.44E-06 | 9.03E-04 | 3.77E-03 | 7.43E-06 | 1.57E-03 | 0.156 | 0.399 |
| Kosakonia | 1.26E-03 | 2.22E-06 | 8.60E-04 | 5.23E-06 | 8.20E-11 | 5.23E-06 | 0.182 | 0.399 |
| Aeromonas | 8.35E-03 | 1.66E-04 | 7.44E-03 | 4.08E-02 | 1.32E-03 | 2.10E-02 | 0.187 | 0.399 |
| Bartonella | 3.10E-02 | 1.47E-03 | 2.21E-02 | 1.23E-03 | 2.28E-06 | 8.72E-04 | 0.204 | 0.399 |
| Tatumella | 2.17E-05 | 3.54E-10 | 1.09E-05 | 5.40E-06 | 8.74E-11 | 5.40E-06 | 0.209 | 0.399 |
| Pectinatus | 2.23E-01 | 6.33E-03 | 4.59E-02 | 1.14E-01 | 1.42E-02 | 6.89E-02 | 0.236 | 0.399 |
| Sebaldella | 1.09E-05 | 8.84E-11 | 5.43E-06 | 0.00E+00 | 0.00E+00 | 0.00E+00 | 0.237 | 0.399 |
| Pseudomonas | 5.15E-03 | 7.91E-05 | 5.14E-03 | 0.00E+00 | 0.00E+00 | 0.00E+00 | 0.324 | 0.464 |
| Enterobacter | 2.59E-03 | 2.00E-05 | 2.58E-03 | 0.00E+00 | 0.00E+00 | 0.00E+00 | 0.329 | 0.464 |
| Salmonella | 1.24E-04 | 4.60E-08 | 1.24E-04 | 0.00E+00 | 0.00E+00 | 0.00E+00 | 0.372 | 0.496 |
| Bombella | 1.20E-01 | 9.30E-03 | 5.57E-02 | 1.91E-01 | 8.38E-03 | 5.28E-02 | 0.487 | 0.591 |
| Apibacter | 1.21E-01 | 2.67E-03 | 2.98E-02 | 8.06E-02 | 3.25E-03 | 3.29E-02 | 0.492 | 0.591 |
| Bifidobacterium | 4.39E-02 | 4.67E-04 | 1.25E-02 | 5.43E-02 | 2.37E-04 | 8.89E-03 | 0.602 | 0.662 |
| Serratia | 2.46E-04 | 1.11E-07 | 1.92E-04 | 1.04E-04 | 2.37E-08 | 8.89E-05 | 0.607 | 0.662 |
| Dysgonomonas | 6.17E-03 | 6.81E-05 | 4.77E-03 | 3.30E-03 | 8.35E-06 | 1.67E-03 | 0.678 | 0.707 |
| Candidatus  _Schmidhempelia | 1.58E-03 | 2.66E-07 | 2.98E-04 | 3.43E-03 | 6.91E-06 | 1.52E-03 | 0.250 | 0.399 |

Note: P value less than 0.05 means significant difference. C (Control) VS TDA (difenoconazole + acetamiprid treated).

**Table S5** Metastats statistical result at genus taxonomy level of gut bacterial microbiota

| genus | Mean  (C) | Variance(C) | Std.err  (C) | Mean  (TD) | Variance  (TD) | Std.err  (TD) | P value | Q value |
| --- | --- | --- | --- | --- | --- | --- | --- | --- |
| Lactobacillus | 1.59E-01 | 5.34E-05 | 4.22E-03 | 2.52E-01 | 1.23E-03 | 2.02E-02 | 0.008 | 0.152 |
| Candidatus  _Schmidhempelia | 1.58E-03 | 2.66E-07 | 2.98E-04 | 9.32E-03 | 1.58E-05 | 2.29E-03 | 0.021 | 0.17 |
| Gilliamella | 1.13E-01 | 2.45E-03 | 2.86E-02 | 2.03E-01 | 6.52E-04 | 1.47E-02 | 0.038 | 0.205 |
| Pectinatus | 2.23E-01 | 6.33E-03 | 4.59E-02 | 7.38E-02 | 4.31E-03 | 3.79E-02 | 0.047 | 0.205 |
| Klebsiella | 6.33E-03 | 2.12E-05 | 2.66E-03 | 1.26E-04 | 4.27E-10 | 1.19E-05 | 0.051 | 0.205 |
| Fructobacillus | 2.01E-02 | 2.72E-04 | 9.53E-03 | 1.48E-04 | 3.70E-08 | 1.11E-04 | 0.061 | 0.207 |
| Escherichia-Shigella | 4.59E-02 | 2.51E-03 | 2.89E-02 | 7.02E-05 | 1.19E-09 | 1.99E-05 | 0.127 | 0.381 |
| Kosakonia | 1.26E-03 | 2.22E-06 | 8.60E-04 | 1.00E-05 | 7.53E-11 | 5.01E-06 | 0.168 | 0.407 |
| Snodgrassella | 8.25E-02 | 1.97E-04 | 8.10E-03 | 6.83E-02 | 1.15E-04 | 6.20E-03 | 0.182 | 0.407 |
| Tatumella | 2.17E-05 | 3.54E-10 | 1.09E-05 | 5.08E-06 | 7.74E-11 | 5.08E-06 | 0.191 | 0.407 |
| Bartonella | 3.10E-02 | 1.47E-03 | 2.21E-02 | 4.47E-04 | 1.69E-07 | 2.37E-04 | 0.204 | 0.407 |
| Serratia | 2.46E-04 | 1.11E-07 | 1.92E-04 | 4.53E-05 | 9.38E-10 | 1.77E-05 | 0.31 | 0.486 |
| Bombella | 1.20E-01 | 9.30E-03 | 5.57E-02 | 1.78E-01 | 1.58E-04 | 7.25E-03 | 0.315 | 0.486 |
| Pseudomonas | 5.15E-03 | 7.91E-05 | 5.14E-03 | 0.00E+00 | 0.00E+00 | 0.00E+00 | 0.34 | 0.486 |
| Enterobacter | 2.59E-03 | 2.00E-05 | 2.58E-03 | 5.08E-06 | 7.74E-11 | 5.08E-06 | 0.344 | 0.486 |
| Salmonella | 1.24E-04 | 4.60E-08 | 1.24E-04 | 0.00E+00 | 0.00E+00 | 0.00E+00 | 0.394 | 0.525 |
| Bifidobacterium | 4.39E-02 | 4.67E-04 | 1.25E-02 | 5.68E-02 | 5.09E-04 | 1.30E-02 | 0.597 | 0.731 |
| Sebaldella | 1.09E-05 | 8.84E-11 | 5.43E-06 | 4.92E-06 | 7.26E-11 | 4.92E-06 | 0.609 | 0.731 |
| Dysgonomonas | 6.17E-03 | 6.81E-05 | 4.77E-03 | 3.27E-03 | 4.68E-07 | 3.95E-04 | 0.648 | 0.74 |
| Aeromonas | 8.35E-03 | 1.66E-04 | 7.44E-03 | 1.47E-02 | 3.32E-04 | 1.05E-02 | 0.706 | 0.759 |
| Atopobium | 6.52E-03 | 2.44E-06 | 9.03E-04 | 8.76E-03 | 7.21E-05 | 4.90E-03 | 0.727 | 0.759 |
| Apibacter | 1.21E-01 | 2.67E-03 | 2.98E-02 | 1.32E-01 | 8.71E-04 | 1.70E-02 | 0.785 | 0.785 |

Note: P value less than 0.05 means significant difference. C (Control) VS TD (difenoconazole treated).

**Table S6** Metastats statistical result at genus taxonomy level of gut bacterial microbiota

| genus | Mean  (C) | Variance(C) | Std.err  (C) | Mean  (TA) | Variance  (TA) | Std.err  (TA) | P value | Q value |
| --- | --- | --- | --- | --- | --- | --- | --- | --- |
| Lactobacillus | 1.59E-01 | 5.34E-05 | 4.22E-03 | 1.00E-01 | 2.19E-04 | 8.54E-03 | 0.00724 | 0.174 |
| Apibacter | 1.21E-01 | 2.67E-03 | 2.98E-02 | 5.91E-02 | 1.54E-05 | 2.26E-03 | 0.049 | 0.588 |
| Candidatus_  Schmidhempelia | 1.58E-03 | 2.66E-07 | 2.98E-04 | 3.27E-03 | 3.51E-06 | 1.08E-03 | 0.122 | 0.693 |
| Kosakonia | 1.26E-03 | 2.22E-06 | 8.60E-04 | 4.39E-05 | 5.70E-10 | 1.38E-05 | 0.142 | 0.693 |
| Bombella | 1.20E-01 | 9.30E-03 | 5.57E-02 | 2.29E-01 | 8.79E-03 | 5.41E-02 | 0.146 | 0.693 |
| Serratia | 2.46E-04 | 1.11E-07 | 1.92E-04 | 1.57E-05 | 7.38E-10 | 1.57E-05 | 0.173 | 0.693 |
| Bifidobacterium | 4.39E-02 | 4.67E-04 | 1.25E-02 | 2.86E-02 | 8.54E-05 | 5.34E-03 | 0.222 | 0.760 |
| Tatumella | 2.17E-05 | 3.54E-10 | 1.09E-05 | 2.01E-03 | 1.02E-05 | 1.85E-03 | 0.281 | 0.843 |
| Sebaldella | 1.09E-05 | 8.84E-11 | 5.43E-06 | 1.77E-02 | 9.42E-04 | 1.77E-02 | 0.376 | 0.895 |
| Pseudomonas | 5.15E-03 | 7.91E-05 | 5.14E-03 | 2.99E-05 | 1.53E-09 | 2.26E-05 | 0.389 | 0.895 |
| Klebsiella | 6.33E-03 | 2.12E-05 | 2.66E-03 | 6.64E-02 | 1.12E-02 | 6.10E-02 | 0.436 | 0.895 |
| Enterobacter | 2.59E-03 | 2.00E-05 | 2.58E-03 | 6.91E-05 | 8.81E-09 | 5.42E-05 | 0.447 | 0.895 |
| Salmonella | 1.24E-04 | 4.60E-08 | 1.24E-04 | 7.33E-06 | 1.61E-10 | 7.33E-06 | 0.526 | 0.909 |
| Snodgrassella | 8.25E-02 | 1.97E-04 | 8.10E-03 | 6.77E-02 | 5.82E-04 | 1.39E-02 | 0.553 | 0.909 |
| Dysgonomonas | 6.17E-03 | 6.81E-05 | 4.77E-03 | 1.10E-02 | 3.43E-05 | 3.38E-03 | 0.606 | 0.909 |
| Panax_ginseng | 5.40E-06 | 8.75E-11 | 5.40E-06 | 1.57E-05 | 7.38E-10 | 1.57E-05 | 0.705 | 0.943 |
| Atopobium | 6.52E-03 | 2.44E-06 | 9.03E-04 | 5.85E-03 | 1.53E-06 | 7.15E-04 | 0.747 | 0.943 |
| Pectinatus | 2.23E-01 | 6.33E-03 | 4.59E-02 | 1.91E-01 | 3.55E-03 | 3.44E-02 | 0.758 | 0.943 |
| Aeromonas | 8.35E-03 | 1.66E-04 | 7.44E-03 | 4.85E-03 | 3.45E-06 | 1.07E-03 | 0.798 | 0.943 |
| Gilliamella | 1.13E-01 | 2.45E-03 | 2.86E-02 | 1.03E-01 | 2.51E-03 | 2.89E-02 | 0.877 | 0.943 |
| Bartonella | 3.10E-02 | 1.47E-03 | 2.21E-02 | 4.03E-02 | 2.72E-03 | 3.01E-02 | 0.881 | 0.943 |
| Escherichia-Shigella | 4.59E-02 | 2.51E-03 | 2.89E-02 | 3.91E-02 | 4.15E-03 | 3.72E-02 | 0.927 | 0.943 |
| Fructobacillus | 2.01E-02 | 2.72E-04 | 9.53E-03 | 2.15E-02 | 4.67E-04 | 1.25E-02 | 0.943 | 0.943 |

Note: P value less than 0.05 means significant difference. C (Control) VS TA (acetamiprid treated).

Table S7 Metastats statistical result at class taxonomy level of gut fungal microbiota

| class | Mean(C) | Variance  (C) | Std.err  (C) | Mean  (TA) | Variance  (TA) | Std.err  (TA) | P value | Q value |
| --- | --- | --- | --- | --- | --- | --- | --- | --- |
| Saccharomycetes | 1.46E-01 | 3.18E-02 | 1.03E-01 | 3.90E-01 | 3.94E-03 | 3.62E-02 | 0.021 | 0.325 |
| Eurotiomycetes | 4.48E-02 | 1.03E-03 | 1.85E-02 | 1.19E-01 | 2.41E-03 | 2.83E-02 | 0.026 | 0.325 |
| Chytridiomycetes | 2.34E-04 | 4.89E-08 | 1.28E-04 | 5.34E-05 | 2.29E-09 | 2.76E-05 | 0.119 | 0.864 |
| Tremellomycetes | 2.49E-02 | 2.20E-04 | 8.56E-03 | 1.43E-02 | 6.20E-06 | 1.44E-03 | 0.173 | 0.864 |
| Olpidiomycetes | 3.31E-04 | 2.88E-07 | 3.10E-04 | 8.92E-06 | 5.97E-11 | 4.46E-06 | 0.268 | 0.864 |
| Mucoromycetes | 0.00E+00 | 0.00E+00 | 0.00E+00 | 1.32E-03 | 5.25E-06 | 1.32E-03 | 0.345 | 0.864 |
| Orbiliomycetes | 2.56E-04 | 1.97E-07 | 2.56E-04 | 0.00E+00 | 0.00E+00 | 0.00E+00 | 0.345 | 0.864 |
| Ustilaginomycetes | 0.00E+00 | 0.00E+00 | 0.00E+00 | 3.68E-04 | 4.07E-07 | 3.68E-04 | 0.345 | 0.864 |
| Wallemiomycetes | 0.00E+00 | 0.00E+00 | 0.00E+00 | 1.64E-03 | 8.05E-06 | 1.64E-03 | 0.345 | 0.864 |
| Agaricostilbomycetes | 7.63E-04 | 1.74E-06 | 7.63E-04 | 4.50E-06 | 6.08E-11 | 4.50E-06 | 0.525 | 0.974 |
| Archaeosporomycetes | 4.59E-06 | 6.33E-11 | 4.59E-06 | 6.44E-04 | 1.24E-06 | 6.44E-04 | 0.550 | 0.974 |
| Pezizomycetes | 9.56E-06 | 2.74E-10 | 9.56E-06 | 6.30E-04 | 1.19E-06 | 6.30E-04 | 0.582 | 0.974 |
| GS35 | 1.32E-03 | 2.73E-06 | 9.54E-04 | 3.80E-04 | 1.11E-08 | 6.08E-05 | 0.608 | 0.974 |
| Malasseziomycetes | 4.69E-03 | 6.24E-05 | 4.56E-03 | 1.13E-03 | 2.06E-07 | 2.62E-04 | 0.697 | 0.974 |
| Agaricomycetes | 1.48E-01 | 1.49E-02 | 7.05E-02 | 9.80E-02 | 2.06E-06 | 8.28E-04 | 0.719 | 0.974 |
| Mortierellomycetes | 1.34E-02 | 9.20E-05 | 5.54E-03 | 2.07E-02 | 2.74E-04 | 9.56E-03 | 0.728 | 0.974 |
| Microbotryomycetes | 4.05E-03 | 2.90E-05 | 3.11E-03 | 6.60E-03 | 2.30E-05 | 2.77E-03 | 0.745 | 0.974 |
| Leotiomycetes | 3.14E-02 | 5.55E-04 | 1.36E-02 | 2.38E-02 | 9.55E-06 | 1.78E-03 | 0.774 | 0.974 |
| Sordariomycetes | 1.98E-01 | 2.24E-02 | 8.64E-02 | 1.51E-01 | 1.17E-04 | 6.24E-03 | 0.779 | 0.974 |
| Dothideomycetes | 2.93E-02 | 1.07E-03 | 1.89E-02 | 3.80E-02 | 1.92E-03 | 2.53E-02 | 0.865 | 0.987 |
| Rhizophydiomycetes | 2.65E-03 | 4.27E-06 | 1.19E-03 | 2.19E-03 | 9.18E-06 | 1.75E-03 | 0.883 | 0.987 |
| Cystobasidiomycetes | 6.98E-04 | 1.46E-06 | 6.98E-04 | 8.55E-04 | 2.19E-06 | 8.55E-04 | 0.908 | 0.987 |

Note: P value less than 0.05 means significant difference. C (Control) VS TA (acetamiprid treated).

Table S8 Metastats statistical result at class taxonomy level of gut fungal microbiota

| class | Mean  (TA) | Variance  (TA) | Std.err  (TA) | Mean  (TDA) | Variance  (TDA) | Std.err  (TDA) | P value | Q value |
| --- | --- | --- | --- | --- | --- | --- | --- | --- |
| Malasseziomycetes | 1.13E-03 | 2.06E-07 | 2.62E-04 | 6.96E-05 | 3.02E-09 | 3.17E-05 | 0.005 | 0.119 |
| Chytridiomycetes | 5.34E-05 | 2.29E-09 | 2.76E-05 | 1.90E-04 | 1.52E-09 | 2.25E-05 | 0.010 | 0.119 |
| Leotiomycetes | 2.38E-02 | 9.55E-06 | 1.78E-03 | 1.44E-02 | 1.34E-05 | 2.11E-03 | 0.014 | 0.119 |
| Eurotiomycetes | 1.19E-01 | 2.41E-03 | 2.83E-02 | 2.72E-02 | 5.68E-05 | 4.35E-03 | 0.024 | 0.119 |
| Microbotryomycetes | 6.60E-03 | 2.30E-05 | 2.77E-03 | 1.42E-04 | 1.79E-08 | 7.73E-05 | 0.045 | 0.178 |
| Sordariomycetes | 1.51E-01 | 1.17E-04 | 6.24E-03 | 1.18E-01 | 5.94E-04 | 1.41E-02 | 0.050 | 0.178 |
| Archaeorhizomycetes | 0.00E+00 | 0.00E+00 | 0.00E+00 | 2.87E-04 | 6.23E-08 | 1.44E-04 | 0.058 | 0.182 |
| Agaricostilbomycetes | 4.50E-06 | 6.08E-11 | 4.50E-06 | 3.34E-04 | 9.65E-08 | 1.79E-04 | 0.085 | 0.236 |
| Mortierellomycetes | 2.07E-02 | 2.74E-04 | 9.56E-03 | 5.88E-03 | 1.96E-06 | 8.08E-04 | 0.117 | 0.292 |
| GS35 | 3.80E-04 | 1.11E-08 | 6.08E-05 | 5.72E-04 | 5.76E-08 | 1.39E-04 | 0.156 | 0.354 |
| Dothideomycetes | 3.80E-02 | 1.92E-03 | 2.53E-02 | 8.48E-03 | 1.43E-05 | 2.18E-03 | 0.199 | 0.393 |
| Tremellomycetes | 1.43E-02 | 6.20E-06 | 1.44E-03 | 1.10E-02 | 1.84E-05 | 2.48E-03 | 0.204 | 0.393 |
| Archaeosporomycetes | 6.44E-04 | 1.24E-06 | 6.44E-04 | 0.00E+00 | 0.00E+00 | 0.00E+00 | 0.306 | 0.403 |
| Cystobasidiomycetes | 8.55E-04 | 2.19E-06 | 8.55E-04 | 0.00E+00 | 0.00E+00 | 0.00E+00 | 0.306 | 0.403 |
| Mucoromycetes | 1.32E-03 | 5.25E-06 | 1.32E-03 | 0.00E+00 | 0.00E+00 | 0.00E+00 | 0.306 | 0.403 |
| Pezizomycetes | 6.30E-04 | 1.19E-06 | 6.30E-04 | 0.00E+00 | 0.00E+00 | 0.00E+00 | 0.306 | 0.403 |
| Ustilaginomycetes | 3.68E-04 | 4.07E-07 | 3.68E-04 | 0.00E+00 | 0.00E+00 | 0.00E+00 | 0.306 | 0.403 |
| Wallemiomycetes | 1.64E-03 | 8.05E-06 | 1.64E-03 | 0.00E+00 | 0.00E+00 | 0.00E+00 | 0.306 | 0.403 |
| Agaricomycetes | 9.80E-02 | 2.06E-06 | 8.28E-04 | 3.02E-01 | 1.52E-01 | 2.25E-01 | 0.595 | 0.723 |
| Rhizophydiomycetes | 2.19E-03 | 9.18E-06 | 1.75E-03 | 6.31E-04 | 1.33E-07 | 2.11E-04 | 0.607 | 0.723 |
| Olpidiomycetes | 8.92E-06 | 5.97E-11 | 4.46E-06 | 6.20E-05 | 1.15E-08 | 6.20E-05 | 0.644 | 0.732 |
| Saccharomycetes | 3.90E-01 | 3.94E-03 | 3.62E-02 | 4.56E-01 | 1.37E-01 | 2.14E-01 | 0.802 | 0.872 |

Note: P value less than 0.05 means significant difference. TA (acetamiprid treated) VS TDA (difenoconazole + acetamiprid treated).

Table S9 Metastats statistical result at class taxonomy level of gut fungal microbiota

| class | Mean  (TD) | Variance  (TD) | Std.err  (TD) | Mean  (TDA) | Variance  (TDA) | Std.err  (TDA) | P value | Q value |
| --- | --- | --- | --- | --- | --- | --- | --- | --- |
| Sordariomycetes | 1.65E-01 | 3.58E-04 | 1.09E-02 | 1.18E-01 | 5.94E-04 | 1.41E-02 | 0.020 | 0.264 |
| Dothideomycetes | 1.14E-01 | 6.10E-03 | 4.51E-02 | 8.48E-03 | 1.43E-05 | 2.18E-03 | 0.034 | 0.264 |
| Malasseziomycetes | 1.33E-03 | 9.38E-07 | 5.59E-04 | 6.96E-05 | 3.02E-09 | 3.17E-05 | 0.039 | 0.264 |
| Eurotiomycetes | 1.12E-01 | 5.16E-03 | 4.15E-02 | 2.72E-02 | 5.68E-05 | 4.35E-03 | 0.053 | 0.264 |
| Mortierellomycetes | 8.34E-03 | 2.87E-06 | 9.79E-04 | 5.88E-03 | 1.96E-06 | 8.08E-04 | 0.076 | 0.317 |
| Leotiomycetes | 1.94E-02 | 8.50E-06 | 1.68E-03 | 1.44E-02 | 1.34E-05 | 2.11E-03 | 0.091 | 0.326 |
| Tremellomycetes | 1.62E-02 | 8.28E-06 | 1.66E-03 | 1.10E-02 | 1.84E-05 | 2.48E-03 | 0.106 | 0.330 |
| Cystobasidiomycetes | 1.73E-03 | 4.55E-06 | 1.23E-03 | 0.00E+00 | 0.00E+00 | 0.00E+00 | 0.146 | 0.405 |
| Microbotryomycetes | 3.77E-03 | 2.55E-05 | 2.92E-03 | 1.42E-04 | 1.79E-08 | 7.73E-05 | 0.199 | 0.496 |
| Agaricostilbomycetes | 1.29E-02 | 4.43E-04 | 1.22E-02 | 3.34E-04 | 9.65E-08 | 1.79E-04 | 0.278 | 0.631 |
| Agaricomycetes | 7.30E-02 | 3.57E-04 | 1.09E-02 | 3.02E-01 | 1.52E-01 | 2.25E-01 | 0.303 | 0.631 |
| Wallemiomycetes | 2.44E-03 | 1.77E-05 | 2.43E-03 | 0.00E+00 | 0.00E+00 | 0.00E+00 | 0.354 | 0.681 |
| Exobasidiomycetes | 3.93E-04 | 4.63E-07 | 3.93E-04 | 0.00E+00 | 0.00E+00 | 0.00E+00 | 0.387 | 0.691 |
| Chytridiomycetes | 8.81E-03 | 2.28E-04 | 8.72E-03 | 1.90E-04 | 1.52E-09 | 2.25E-05 | 0.487 | 0.793 |
| Olpidiomycetes | 4.52E-06 | 6.12E-11 | 4.52E-06 | 6.20E-05 | 1.15E-08 | 6.20E-05 | 0.511 | 0.793 |
| Archaeorhizomycetes | 3.70E-03 | 4.12E-05 | 3.70E-03 | 2.87E-04 | 6.23E-08 | 1.44E-04 | 0.539 | 0.793 |
| Rhizophydiomycetes | 4.85E-04 | 2.67E-08 | 9.43E-05 | 6.31E-04 | 1.33E-07 | 2.11E-04 | 0.717 | 0.995 |
| Saccharomycetes | 3.78E-01 | 2.66E-02 | 9.42E-02 | 4.56E-01 | 1.37E-01 | 2.14E-01 | 0.806 | 1.000 |
| GS35 | 5.49E-04 | 6.01E-08 | 1.41E-04 | 5.72E-04 | 5.76E-08 | 1.39E-04 | 0.905 | 1.000 |
| Archaeosporomycetes | 4.52E-06 | 6.12E-11 | 4.52E-06 | 0.00E+00 | 0.00E+00 | 0.00E+00 | 1.000 | 1.000 |

Note: P value less than 0.05 means significant difference. TD (difenoconazole treated) VS TDA (difenoconazole + acetamiprid treated)


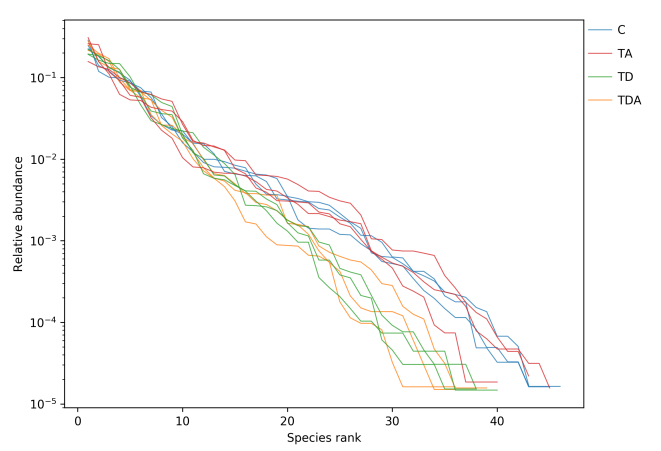


B


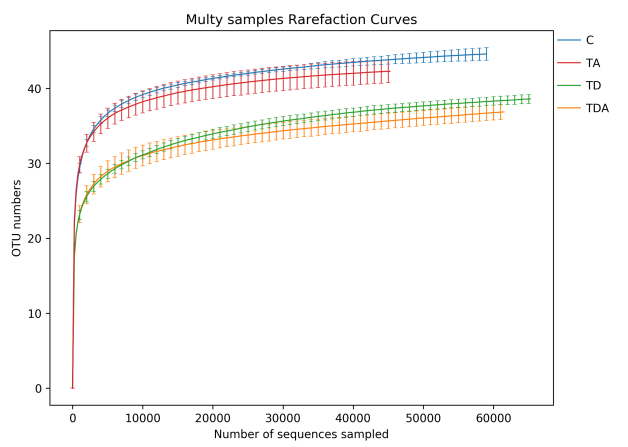


A


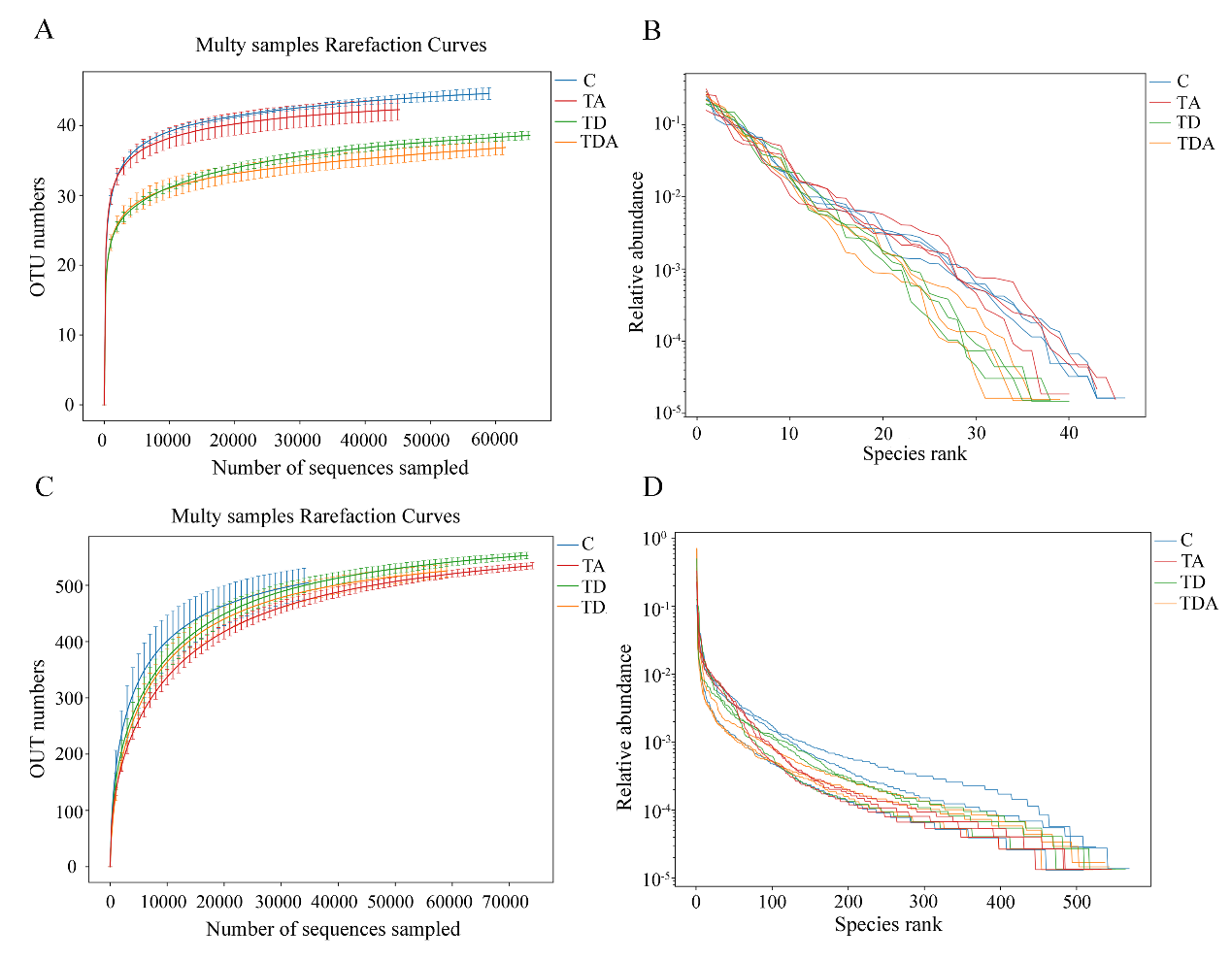


Fig.S1 Effects of acetamiprid or/and difenoconazole on gut bacterial (A and B) and fungal (C and D) microbiota. OUT rank curves (A and C) of gut microbiota for each group and rarefaction curves (B and D) of gut microbiota for each samples, respectively.

C


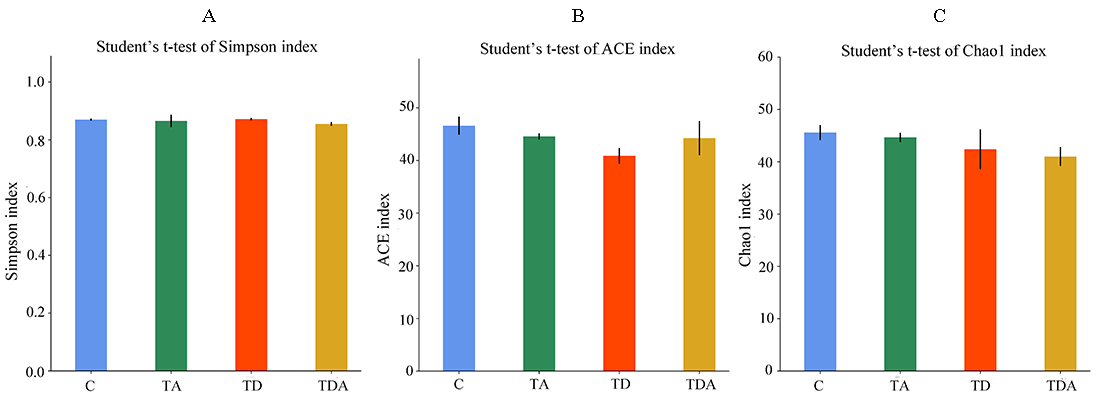


Fig.S2 Alpha diversity of gut bacteria measured by the Simpson (A), ACE (B) and Chao1 (C) indices. Data (mean ± SEM) were analyzed by Student’s t-test (* *P* < 0.05).


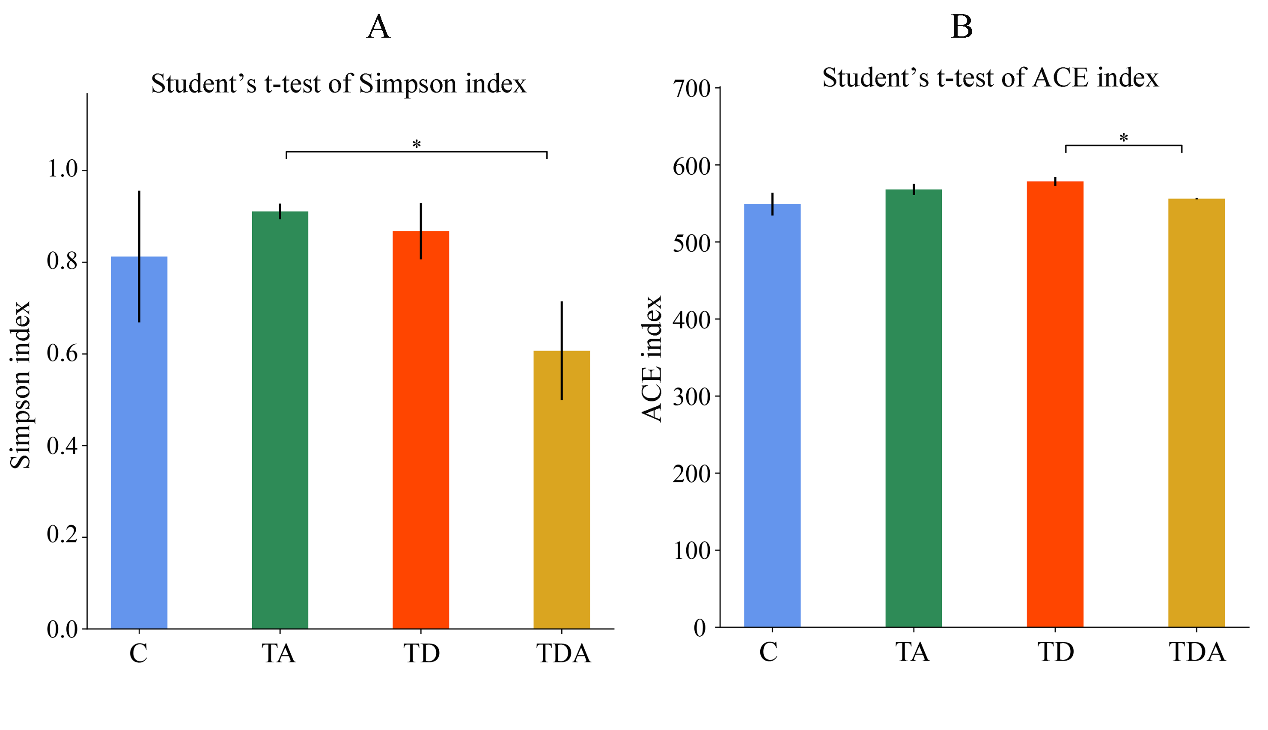


Fig.S3 Alpha diversity of gut fungi measured by the Simpson (A) and ACE (B) indices. Data (mean ± SEM) were analyzed by Student’s t-test (* *P* < 0.05).

S1 Reference

Wu, Y., Zheng, Y., Chen, Y., Chen, Y., Chen, G., Zheng, G., Hu, F., 2020. *Apis cerana* gut microbiota contribute to host health though stimulating host immune system and strengthening host resistance to *Nosema ceranae*. R. Soc. Open Sci. 7: 192100.

Shi, W., Sun, J., Xu, B., Li, Han., 2013. Molecular characterization and oxidative stress response of a cytochrome P450 gene (*CYP4G11*) from *Apis cerana cerana*. Z. Naturforsch. 68 c: 509 - 521.

Jiang, W., He, X., Wang, Z., Yan, W., Zeng, Z., Wu, X., 2016. Cloning and expression analysis of cytochrome CYP9E2 gene in the Chinese honeybee, *Apis cerana cerana*. Acta Entomologica Sinica, 59(10): 1050 - 1057.

Zhu, M., Zhang, W., Liu F., Chen, X., Li, H., Xu, B., 2016. Characterization of an *Apis cerana cerana* cytochrome P450 gene (*AccCYP336A1*) and its roles in oxidative stresses responses. Gene, 584:120 - 128.

Ma, M., Jia, H., Cui, X., Zhai, N., Wang, H., Guo, X., Xu, B., 2018. Isolation of carboxylesterase (esterase FE4) from *Apis cerana cerana* and its role in oxidative resistance during adverse environmental stress. Biochimie, 85-97.

Chao, Y., Wang, C., Jia, H., Zhai, N., Wang, H., Xu, B., Li, Han., Guo, X., Identification of an *Apis cerana cerana* MAP kinase phosphatase 3 gene (*AccMKP3*) in response to environmental stress. Cell Stress Chaperon., 24(6): 1137 - 1149.
